# Unlocking the adaptation mechanisms of the oleaginous microalga *Scenedesmus* sp. BHU1 under elevated salt stress: a physiochemical, lipidomics and transcriptomics approach

**DOI:** 10.3389/fmicb.2024.1475410

**Published:** 2024-11-18

**Authors:** Rahul Prasad Singh, Priya Yadav, Himani Sharma, Ajay Kumar, Abeer Hashem, Elsayed Fathi Abd_Allah, Rajan Kumar Gupta

**Affiliations:** ^1^Laboratory of Algal Research, Department of Botany, Institute of Science, Banaras Hindu University, Varanasi, India; ^2^Amity Institute of Biotechnology, Amity University, Noida, India; ^3^Botany and Microbiology Department, College of Science, King Saud University, Riyadh, Saudi Arabia; ^4^Plant Production Department, College of Food and Agricultural Sciences, King Saud University, Riyadh, Saudi Arabia

**Keywords:** *Scenedesmus* sp., salt stress, chlorophyll a fluorescence, JIP-test, linear electron flow, enzymatic assay, lipidomics, transcriptomics

## Abstract

Microalgae are vital for their photosynthetic abilities, contributing significantly to global oxygen production, serving as a key trophic level in aquatic ecosystems, aiding in biofuel production, assisting in wastewater treatment, and facilitating the synthesis of valuable biochemicals. Despite these advantages, photosynthetic microalgae are sensitive to salt stress, which alters their physiochemical and metabolic status, ultimately reducing microalgal growth. This sensitivity highlights the importance of understanding the impact of elevated salt content on the physiochemical, metabolic, and transcriptomic profiling of *Scenedesmus* sp., areas that are not yet fully understood. Our findings indicate that elevated salt stress decreases photosynthetic efficiency and increases non-regulated photochemical quenching of photosystem II (PSII). Moreover, PSII-driven linear electron flow (LEF) decreased, whereas photosystem I (PSI)-driven cyclic electron flow (CEF) increased in salt-stressed cells. To better understand the electron flow from PSII to PSI under elevated salt treatment, we analyzed the excitation energy flux per reaction center (RC), per cross-section (CS), energy flux ratios, and the potential index of PSII. Additionally, flow cytometry graphs depict the viability assay of *Scenedesmus* sp. BHU1. Our observations further revealed an increase in biochemical attributes, such as stress biomarkers, osmoprotectants, and enzymatic antioxidants, which help scavenge reactive oxygen species (ROS) under salt stress. Intracellular cations (Na ^+^ and Ca^2+^) were increased, while K^+^ levels decreased, indicating mechanisms of cellular homeostasis under salt stress. UHPLC-HRMS-based lipidome analysis confirmed that increasing salt stress induces the hyperaccumulation of several fatty acids involved in adaptation. Moreover, transcriptome analysis revealed the upregulation of genes associated with PSI, glycolysis, starch metabolism, sucrose metabolism, and lipid accumulation under salt stress. In contrast, genes related to PSII and C3 carbon fixation were downregulated to mitigate the adverse effects of salt stress.

## Highlights

- Under enhanced salt stress, the quantum yield of PSII decreased while non-regulated photochemical quenching increased.
- The drop in the OJIP transient curve of stressed cells indicates the oxidative effect of salt, which causes a decline in the electron donor and acceptor pools of PSII.
- PSII-driven LEF reduction and activation of NAD(P)H dehydrogenase-like complex type-1 (NDH-1) based CEF around the PSI were an adaptation strategy to guard PSII from salt-induced photoinhibition.
- In stressed cells, stress biomarkers, osmoprotectants, and enzymatic antioxidants increased to scavenge ROS, while Na^+^ and Ca^2+^ rose and K^+^ dropped to maintain cellular homeostasis.
- UHPLC-HRMS-based lipidomics analysis confirmed the hyperaccumulation of monounsaturated fatty acids (MUFAs) and polyunsaturated fatty acids (PUFAs) in stressed cells, which mitigates the adverse effects of elevated salt.
- Transcriptome profiling reveals the upregulation of PSI and lipid metabolism in *Scenedesmus* sp., highlighting its potential survival mechanisms under salt stress.

## 1 Introduction

Microalgae serve as primary producers of organic matter in aquatic ecosystems and have several advantages over the higher plants due to their higher photosynthetic potential and shorter life span (Singh et al., 2023a; Mehariya et al., 2024). For current and future human needs, microalgae have shown a wide range of applications in various fields, serving as a source of food, antioxidants, fatty acids, hormones, pharmaceuticals, cosmetics, pigments, vitamins, and bioenergy (Grubišić et al., 2019; Anandraj et al., 2020). Increased salinity reduces the biomass of freshwater microalgae but simultaneously increases the production of several important macromolecules and micromolecules, which could be highly beneficial for human welfare. Research on adaptation responses to elevated salinity has primarily focused on marine microalgae, with limited studies available on freshwater microalgae. Hence, it is important to understand how freshwater microalgae acclimate to increased salt levels by altering their physiological and biochemical pathways.

Similar to terrestrial plants, microalgae have the capacity to thrive in brackish or saline water and can effectively utilize wastewater as a nutrient source (Mountourakis et al., 2023). However, high salinity levels induce osmotic, ionic, and oxidative stress, leading to the production of ROS. While ROS serve as secondary messengers in intracellular signaling pathways, excessive accumulation triggers a series of adaptive responses in microalgae to preserve cellular homeostasis. These responses include alterations in photosynthesis, damage to macromolecules (such as proteins and nucleic acids), adjustments in membrane permeability, and acceleration in the synthesis of osmoprotectants (Singh et al., 2023b). Notably, among these factors, photosynthesis emerges as a highly vulnerable target of salinity stress. It is well known that the thylakoid membrane contains two photosystems (PSII and PSI), which are responsible for the light-driven reactions of photosynthesis. These thylakoid membranes generate a proton motive force (PMF), which drives the formation of ATP used in the CO_2_ fixation pathway. Salinity stress interferes with the compositional and operational components of photosynthesis, disrupting the redox equilibrium in microalgal cells. Both osmotic and ionic stresses induced by salinity affect the photosynthesis of microalgal cells, causing alterations in PSII and PSI activity (Allakhverdiev and Murata, 2008). Salt stress results in irregularities in the arrangement of the thylakoid membrane, poor grana stacking, and increased degradation of the D1 protein in the chloroplasts (Yang et al., 2020). Salinity stress triggers the synthesis of ROS in chloroplasts of algae, which inhibits the LEF of PSII (Swapnil et al., 2015).

Moreover, salt-induced stress disturbs the oxygen-evolving complex (OEC) within PSII, leading to a compromised flow of electrons between the oxidizing and reducing sides of the PSII RC. Contrarily, under increased salinity stress, CEF around PSI is increased. It is possible that increased salinity triggers specific signaling pathways that enhance PSI activity as a protective response.

Additionally, PSI plays a crucial role in scavenging ROS, protecting the photosynthetic structure, and maintaining photosynthetic efficiency. During CEF, PSI electrons are redirected back to PSI through the Cyt b_6_f complex and the plastoquinone (PQ) pool. ATP synthesis occurs during CEF due to the proton gradient established across the thylakoid membrane. Consequently, CEF plays a vital role in photoprotection and sustaining ATP levels (Srivastava et al., 2021).

High salt concentrations in the medium hinder the uptake of water and nutrients, thus impeding growth and potentially leading to death. Ionic stress induced by salt arises from an imbalance in ion homeostasis, where salinity causes Na^+^ to compete with K^+^ for uptake, resulting in K^+^ deficiency in the cytosol. High salt levels in the medium decrease osmotic potential and reduce water uptake. To mitigate the damage caused by salt stress, microalgae have evolved various physiological, metabolic, and molecular mechanisms. These mechanisms involve accumulating osmoprotectants such as sucrose, trehalose, and lipids as storage molecules to ensure the survival of microalgae (Yang et al., 2023).

Additionally, microalgae accumulate ROS-scavenging enzymes such as superoxide dismutase (SOD), catalase (CAT), ascorbate peroxidase (APX), peroxidase (POD), glutathione peroxidases (GPX), and glutathione reductase (GR). ROS detoxification also occurs through low molecular weight antioxidants such as ascorbate, glutathione, and polyphenols and by producing compatible solutes such as proline, glycine betaine, and other metabolites to facilitate osmotic adjustment. Ca^2+^ acts as a secondary messenger, initiating a phosphorylation cascade that activates key stress-responsive genes, facilitating algae adaptation and helping them cope with adverse conditions.

Notably, different freshwater algal species exhibit varying degrees of salt tolerance, and the mechanisms underlying their ability to withstand salt stress can differ significantly. Therefore, further research is warranted to better understand what determines the salt tolerance of various freshwater algal species. Hence, significant gaps persist in our knowledge, particularly regarding how salinity-induced stress impacts the electron transport process from PSII to PSI and alters metabolic pathways in freshwater algal species. To address these knowledge gaps, we assessed the effects of salinity stress on the photosynthetic activity, biochemical status, lipidomics, and transcriptomics of *Scenedesmus* sp. BHU1.

## 2 Materials and methods

### 2.1 Experimental organism and growth conditions

A unicellular, non-motile colonial green microalga, *Scenedesmus* sp. BHU1, has been chosen as the experimental organism for this study (Singh et al., 2022). During the experiment, *Scenedesmus* sp. BHU1 was grown in BG-11 N^+^ culture at pH 7.4. The culture was illuminated with 80 μmol photon m^−2^ s^−1^ light provided by a fluorescent cool tube light (20 W, Havells, New Delhi, India), with a 12-h light and 12-h dark photoperiod at 25 ± 2°C. All culture media and glassware were sterilized for 20 min at 15 Lbf/m^2^ pressure at 121°C.

### 2.2 Experimental design and doses of the salt stress

Under the above growth conditions, before the experiment, the axenic mother culture of the *Scenedesmus* sp. BHU1 was grown in a 2 L conical flask containing 1 L of fresh BG-11 N^+^ medium. Each experiment was performed in three biological replicates (*n* = 3) in 250 mL borosil conical flasks containing 100 mL of *Scenedesmus* sp. BHU1 culture of 0.1 optical density (OD) from an aseptically growing mother culture (OD = 0.7) according to the N_1_V_1_ = N_2_V_2_ formula, where N_1_ = optical density (OD = 0.7) of the mother culture, V_1_ = desired volume required, N_2_ = desired optical density (OD = 0.1) and V_2_ = volume of the experimental culture (100 mL).

Regarding the salinity stress doses, freshly prepared 0.05, 0.1, 0.15, 0.2, and 0.4 M NaCl (Merck, Mumbai, India, ≥ 99% purity) solution was sterilized by filtering using the 0.22 μm pore size sterile Millipore membrane filter. The microalgal cells grown in BG-11 N^+^ medium without NaCl were regarded as the control cells (0 M NaCl), while the microalgal cells grown in BG-11 N^+^ medium supplemented with 0.05, 0.1, 0.15, 0.2, and 0.4 M NaCl for 14 days were considered as salt-treated cells. For each treatment (0, 0.05, 0.1, 0.15, 0.2, and 0.4 M NaCl), we prepared three 250 mL conical flasks. The conical flasks were labeled as follows: 0R1, 0R2, and 0R3 for the 0 M NaCl treatment (control); 0.05R1, 0.05R2, and 0.05R3 for the 0.05 M NaCl treatment; 0.1R1, 0.1R2, and 0.1R3 for the 0.1 M NaCl treatment; 0.15R1, 0.15R2, and 0.15R3 for the 0.15 M NaCl treatment; 0.2R1, 0.2R2, and 0.2R3 for the 0.2 M NaCl treatment; and 0.4R1, 0.4R2, and 0.4R3 for the 0.4 M NaCl treatment. To avoid the attachment of microalgal cells to the flask walls, the culture flasks were manually agitated 4–5 times per day.

### 2.3 Assessment of microalgal growth curve and morphology

The growth of *Scenedesmus* sp. BHU1 was assessed by recording the OD at 750 nm using a UV–VIS spectrophotometer (UV-2600, SHIMADZU, Japan). The images were captured at 40X magnification using a light microscope (CX21iLEDFS1, Olympus).

### 2.4 Measurement of physiological attributes

The PAM 2500 fluorometer (Heinz Walz, Effeltrich, Germany) was used to study the photosynthesis of *Scenedesmus* sp. BHU1 based on chlorophyll fluorescence (ChlF) signals. It was connected to a laptop operating data collection software, PamWin 3.20. The chlorophyll content of the microalgal cells from control to 0.4 M NaCl was adjusted to ~6.24 μg/mg. Before taking any measurements, *Scenedesmus* sp. BHU1 cultures were dark-adapted for 10–15 min. The aliquots of dark-adapted microalgal cells were pipetted into a steel cuvette with a tiny magnetic stirrer that homogenized the cells during the entire measurement period.

#### 2.4.1 Measurement of photosynthetic parameters of slow kinetics using ChlF under saturation pulse (SP) mode

The ChlF yield has been measured using a light-emitting diode (LED) delivering modulated actinic light (AL) for the slow kinetics measurement. The dark-adapted minimum ChlF yield (Fo) was determined by delivering low measuring light (ML) intensities (<< 0.1 μmol photons m^−2^ s^−1^), and the dark-adapted maximum ChlF yield (Fm) was determined by a 0.3 s saturating pulse at 3,000 μmol photons m^−2^ s^−1^. Then, a red AL intensity of 104 μmol photons m^−2^ s^−1^ was applied to stimulate photochemistry and enable it to reach a steady state (F_S_). A second saturating pulse (3,000 μmol photons m^−2^ s^−1^) has been delivered during the initiation phase to record the light-adapted maximal ChlF yield (Fm′). Upon completing the induction period and promptly switching off the AL, the light-adapted minimal ChlF yield (Fo′) was measured under 5 μmol photons m^−2^ s^−1^ of far-red irradiation. The PamWin 3.20 software automatically calculated various parameters such as Fv/Fm, Y(II), Fv, Y(NO), Y(NPQ), NPQ, qN, qP, qL, qE, qI, and qNP based on the measured values of Fo, Fm, Fo′, Fm′, and Fs. The definitions and formulas of all these photosynthetic parameters of slow kinetics are provided in Supplementary Table S1. The PamWin 3.20 software used the equations derived from earlier investigations shown in Supplementary Table S1 to calculate the above photosynthetic parameters.

#### 2.4.2 Measurement of photosynthetic parameters of the rapid light curve (RLC) using ChlF under SP mode

*Scenedesmus* sp. BHU1 cells were exposed to a light illumination increase (every 30 s) from 0 to 630 μmol photons m^−2^ s^−1^ of photosynthetically active radiation (PAR). This allowed the determination of the rapid light response curve (α), maximum electron transfer rate (ETR_max_), and minimum saturating light intensity (I_k_). The RLC photosynthetic parameters were measured using the electron transport rate (ETR) versus AL. The PAM device automatically calculated the α, ETR_max_, and I_k_ parameters using the equations provided in Supplementary Table S1.

#### 2.4.3 Measurement of photosynthetic parameters of fast kinetics using ChlF under fast acquisition mode

The ChlF emission generated by the fast acquisition was measured for 1 s between 10 μs and 320 ms at a saturating light pulse of 3,000 μmol photons m^−2^ s^−1^ was applied using the Kautsky transient effect. To calculate various PSII photochemistry parameters, each OJIP transient curve was analyzed using the original data based on the JIP test. The original data included the minimum ChlF intensity (F_O_, O-level), the maximum ChlF intensity (F_M_, M-level), the ChlF intensity at 0.3 ms (F_K_, K- level), the ChlF intensity at 2 ms (F_J_, J-level), and the ChlF intensity at 30 ms (F_I_, I-level). The OJIP transient curve was plotted using the original fluorescence intensities [Ft (V)] data on a logarithmic time scale. It was possible to compute the PSII functional alterations of *Scenedesmus* sp. BHU1 using various fundamental and phenomenological fluorescence data. The definitions and formulas of various photosynthetic attributes derived from the JIP-test to analyze the OJIP fluorescence transient are provided in Supplementary Table S2.

#### 2.4.4 Measurement of post-illumination increase of chlorophyll fluorescence transient (CEF around PSI) under SP mode

CEF was measured by observing the transient increase in the dark level of ChlF after AL illumination. *Scenedesmus* sp. BHU1 cells were exposed to AL (104 μmol photons m^−2^ s^−1^) for 120 s. After that, AL was switched off, and only ML remained on for another 210 s, during which the transient increase in amplitude of ChlF was measured. The increase in amplitude of ChlF after turning off AL is used as a CEF signal around PSI (Srivastava et al., 2021).

### 2.5 Measurement of biochemical attributes

#### 2.5.1 Viability assay

To assess the vitality of *Scenedesmus* sp. BHU1 cells, we used fluorescein diacetate (FDA) dye. FDA is a synthetic, cell-permeable ester—an uncharged, non-fluorescent, lipid-soluble dye. After uptake, it undergoes hydrolysis to fluorescein by intracellular esterases, thereby staining live cells. The fluorescence emitted by fluorescein indicates cellular esterase activity, providing a measure of cell viability. The stock solution of FDA was prepared by dissolving 1 mg of FDA in 1 mL of chilled acetone. Then, 2 μL/mL of the microalgal suspension was used to stain live cells. The stained cells were rinsed with phosphate-buffered saline (PBS) to remove excess dye and then analyzed using flow cytometry (CytoFLEX LX, Beckman Coulter, BC47041, United States). We used 488 nm excitation and 530 nm emission filters in the green channel for the FDA dye. These excitation and emission wavelengths are within the range of the CytoFLEX Channel B525-FITC-A, which utilizes a green 525/40 BP (bandpass) filter. The data from the flow cytometry were analyzed using the software CytExpert 2.3 (Beckman Coulter, Pasadena, CA, United States).

#### 2.5.2 Assessment of ROS and osmoprotectant production

To evaluate ROS levels in *Scenedesmus* sp. BHU1, a highly sensitive dye, 2,7-dichlorodihydrofluorescein diacetate (DCFH-DA), was used. The microalgal suspension was treated with 5 μM DCFH-DA and incubated in the dark at room temperature for 45 min. Following incubation, the cells were thoroughly washed with 50 mM PBS. In the presence of ROS, the DCFH dye is oxidized to the highly fluorescent DCF. The stained cells were then examined using a fluorescent spectrophotometer (Agilent Technologies, Cary Eclipse) with an excitation wavelength of 485 nm and emission detected between 500 and 600 nm.

The sucrose content in *Scenedesmus* sp. BHU1 was determined using the method described by Van Handel (1968). *Scenedesmus* sp. BHU1 cells were mixed with 3 mL of 80% ethanol and heated in a water bath at 100°C until the alcohol had completely evaporated. Then, 0.1 mL of 30% aqueous KOH was added to the residue, and the mixture was allowed to stand at 100°C for 10 min. After cooling to room temperature, 3 mL of anthrone reagent (1.5% anthrone in 70% chilled H_2_SO_4_) was added, and the mixture was warmed to 40°C for 15 min. Once cooled, the absorbance was measured at 620 nm using a UV–VIS spectrophotometer, and the sucrose content was calculated in μg mg^−1^ FW using a sucrose calibration curve.

The trehalose content in *Scenedesmus* sp. BHU1 was determined using the method outlined by Lillie and Pringle (1980). *Scenedesmus* sp. BHU1 cells were homogenized in 3 mL of 500 mM trichloroacetic acid and centrifuged at 6,000 rpm for 5 min to collect the supernatant. One mL of the supernatant was rapidly chilled in an ice bucket, followed by the addition of 5 mL of chilled anthrone reagent. The tubes were incubated at 90°C in a water bath for 5 min and then allowed to cool to room temperature. The absorbance of the sample was then measured at 625 nm using a UV–VIS spectrophotometer. The amount of trehalose was determined in μg mg^−1^ FW using a trehalose calibration curve.

#### 2.5.3 Measurement of intracellular cations and cations ratio

Intracellular cations were quantified using the tri-acid technique, following the method outlined by Allen et al. (1986) with minor modifications. 50 mg of lyophilized biomass of *Scenedesmus* sp. BHU1 was combined with a mixture of concentrated HNO₃, HClO₄, and H₂SO₄ (5:1:1, v/v/v). The mixture was left to react overnight. The resulting solution was then heated on a hot plate until it became colorless. After cooling to room temperature, the solution was diluted to a suitable volume and filtered using Whatman filter paper. The atomic absorption spectrophotometer (AAS; PerkinElmer Analyst 800, United States) was used to analyze cation content.

#### 2.5.4 Assay of enzymatic antioxidant molecules

*Scenedesmus* sp. BHU1 cells were homogenized in a 2 mL extraction buffer containing 100 mM phosphate buffer (pH 7.5) and 0.5 mM EDTA. After centrifugation at 10,000 rpm for 20 min at 4°C, the resulting crude extract was used for the enzymatic assay.

SOD activity was evaluated using the method described by Giannopolitis and Ries (1977). For the SOD assay, a 3 mL reaction mixture was prepared, consisting of 1.5 mL of 50 mM phosphate buffer (pH 7.5), 0.2 mL of 13 mM methionine, 0.1 mL of 75 μM NBT, 0.1 mL of 0.1 mM EDTA, and 0.1 mL of the enzyme extract. Then, 0.1 mL of 2 μM riboflavin was added, and the volume was adjusted to 3 mL with distilled water. This mixture was exposed to light (100 μmole photon m^−2^ s^−1^) for 10 min, and the absorbance at 560 nm was recorded using a UV–VIS spectrophotometer. A non-irradiated reaction mixture served as the blank. One unit of SOD activity was quantified as the amount of enzyme required to induce 50% inhibition in the reduction of NBT.

CAT activity was evaluated using the method outlined by Aebi (1984). The reaction mixture comprised 1.5 mL of 50 mM phosphate buffer (pH 7.0), 0.5 mL of 30 mM H_2_O_2_, 0.1 mL enzyme extract, and distilled water to reach a final volume of 3 mL. After thoroughly mixing the reaction mixture, absorbance was measured at 240 nm using a UV–VIS spectrophotometer. CAT activity was determined using an extinction coefficient of 0.0436 mM^−1^ cm^−1^ and expressed as nM mg^−1^ FW min^−1^.

APX activity was assessed using the procedure outlined by Nakano and Asada (1981). Following centrifugation at 10,000 rpm for 20 min at 4°C, the resulting supernatant was used for the APX assay. The reaction mixture, with a final volume of 3 mL, consisted of 1.5 mL of 50 mM phosphate buffer (pH 7.0), 0.1 mL of 1 mM EDTA, 0.5 mL of 0.5 mM ascorbic acid, 0.1 mL of 0.1 mM H_2_O_2_, and 0.7 mL distilled water. Subsequently, 0.1 mL of enzyme extract was added to the reaction mixture. The oxidation of ascorbic acid was monitored by measuring the reduction in absorbance at 290 nm using a UV–VIS spectrophotometer. The calculation used an extinction coefficient of 2.8 mM^−1^ cm^−1^, and enzyme activity was expressed as nM mg^−1^ FW min^−1^.

POD activity was determined using the method described by Gahagan et al. (1968). The total volume of the reaction mixture was 3 mL, comprising 0.8 mL of 100 mM phosphate buffer (pH 7.0), 1 mL of 100 mM pyrogallol, 1 mL of 25 mM H_2_O_2_, and 0.2 mL of enzyme extract. Following thorough mixing, absorbance was measured at 430 nm using a UV–VIS spectrophotometer. The calculation used an extinction coefficient of 12 mM^−1^ cm^−1^, and enzyme activity was expressed as nM mg^−1^ FW min^−1^.

GPX activity was assayed by measuring the oxidation of guaiacol to tetraguaiacol, as Castillo et al. (1984) described. The 3 mL reaction mixture contained 1.5 mL of 50 mM phosphate buffer (pH 6.1), 0.5 mL of 16 mM guaiacol, 0.5 mL of 2 mM H_2_O_2_, 0.1 mL of crude enzyme extract, and 0.4 mL of distilled water. Following thorough mixing, absorbance was measured at 470 nm using a UV–VIS spectrophotometer. The calculation used an extinction coefficient of 26.2 mM^−1^ cm^−1^, and enzyme activity was expressed as nM mg^−1^ FW min^−1^.

GR activity was assessed following the method outlined by Smith et al. (1988). For the GR assay, a 2 mL reaction mixture was prepared, comprising 1 mL of 66.67 mM phosphate buffer (pH 7.5, containing 0.33 mM EDTA), 0.5 mL of 0.5 mM DTNB, 0.1 mM of 66.67 mM nicotinamide adenine dinucleotide phosphate hydrogen (NADPH), 0.1 mL of 0.66 mM GSSG, and 0.3 mL of distilled water. Enzyme activity was determined by monitoring the increase in absorbance of NADPH at 412 nm using a UV–VIS spectrophotometer. Calculations were performed using an extinction coefficient of 6.22 mM^−1^ cm^−1^, and enzyme activity was expressed as nM mg^−1^ FW min^−1^.

### 2.6 Extraction of metabolites and their profiling using UHPLC-HRMS

For metabolic profiling, metabolites were extracted from control (0 M NaCl) and 0.4 M NaCl-treated cells using HPLC-grade solvents. 100 mg lyophilized algal biomass was homogenized in methanol, ethyl acetate, and chloroform (40:30:30 v/v/v). The homogenized samples were placed on a rotary shaker at 100 rpm for 24 h. Subsequently, the samples were centrifuged at 10,000 rpm for 20 min at 4°C, and the supernatants were collected. This process was repeated three times for complete metabolite extraction until the pellet turned white. The supernatants were then evaporated using an IKA MC3i rotary vacuum evaporator, and the samples were dried with a Welch dry vacuum pump. Following this, the crude extract of metabolites was dissolved in 2 mL of HRMS-grade methanol and mixed thoroughly. The solution was then filtered through a 0.22 μm pore size Millipore membrane filter.

The profiling of filtrate was carried out using UHPLC-HRMS (Orbitrap Eclipse Tribrid Mass Spectrometer, United States). A three-solvent system was used as the mobile phase: solvent A comprised water with 0.1% formic acid, solvent B consisted of 100% acetonitrile with 0.1% formic acid, and solvent C was 100% methanol with 0.1% formic acid. Metabolites were separated using a GOLD C18 selectivity HPLC column (2.1 mm inner diameter, 100 mm length, and 1.9 μm particle size). The injection volume was 5 μL, the runtime lasted for 30 min, and the flow rate was maintained at 0.3 mL/min. Following separation, the column outlet was connected to a mass spectrometer via H-ESI (electrospray ionization). Compounds were ionized in both negative and positive modes using H-ESI and detected with the Orbitrap. Identification of likely compounds in the extract involved comparing the MS spectra of analyzed samples with those from Predicted Compositions, mzCloud Search, ChemSpider Search, and MassList Search databases.

### 2.7 Transcriptome analysis

#### 2.7.1 Transcriptome assembly of raw sequencing reads

Transcriptome assembly was performed using Illumina reads, and MultiQC (v.1.13) was used to assess the quality of the raw reads. Following the quality assessment, the quality-trimmed reads were assembled into draft contigs. For this purpose, the Trinity assembler was used with different kmer lengths (from 21 bp to 127 bp) to generate the contigs and scaffolds (“trinity_out_dir.Trinity.fasta”) in the main directory. Large amounts of RNA-seq reads were processed by Trinity using three separate software modules: Inchworm, Chrysalis, and Butterfly, which were deployed in a sequential manner. To separate transcripts originating from paralogous genes and extract full-length splicing isoforms, Trinity divides the sequencing data into numerous distinct de Bruijn graphs, each representing the transcriptional complexity at a particular gene or location.^1^ After completing the assembly, the quality of the newly assembled transcriptome was assessed using the following methods. First, RNA-seq reads were aligned to the transcriptome using Bowtie2, and the numbers of proper pairs and improper or orphan read alignments were counted with Samtools. Next, to ensure the presence of full-length transcripts, the assembled transcripts were aligned to the SwissProt database using BlastX. Finally, raw reads were aligned to the transcript assembly using the Bowtie2 alignment-based technique in conjunction with RSEM for transcript quantification.

#### 2.7.2 Structural and functional annotations

TransDecoder identifies putative coding regions within transcript sequences, such as those generated by Trinity’s RNA-Seq transcript assembly. TransDecoder was used to process the full transcriptome of *Scenedesmus* sp. BHU1 uses thousands of transcript sequences as input. A comprehensive set of annotation tools called Trinotate is used to automatically and functionally annotate transcriptomes. The Trinotate pipeline was utilized for functional analysis and enrichment analysis using ClusterProfiler. Trinotate employs several well-established techniques for functional annotation, such as protein domain identification (HMMER/PFAM), homology search against known sequence data (BLAST+/SwissProt), prediction of transmembrane domains and signal peptides in proteins (SignalP/tmHMM), and utilization of multiple annotation databases (eggNOG/GO/KEGG). The entire set of functional annotation data obtained from transcript analysis was combined into an SQLite database, enabling quick and effective searches for terms associated with desired characteristics in the annotation report for the transcriptome as a whole. Following structural and functional annotation, gene names were appended to the gene result files produced during the quantification phase. Before calculating differential expression, we matched the gene names across the samples. For estimating differential gene expression (DGE), edgeR employed raw counts with a 0.05 *p*-value threshold.

### 2.8 Statistical analysis

The results were presented using the average values from three biological replicates (*n* = 3) and the standard errors of the mean (SEM). Scientific data analysis was performed using SPSS 21.0 (IBM Corp., New York, United States), applying one-way analysis of variance (ANOVA) to identify the significant effects of salinity stress and its interactions on *Scenedesmus* sp. BHU1. Tukey’s *post hoc* test, with a probability threshold of *p* < 0.05, was used to determine significant differences among treatments. Our study involves comparing more than two groups, so ANOVA is typically used to determine if there are statistically significant differences between the group means. However, while ANOVA identifies the presence of differences, it does not specify which groups differ. To address this, a *post hoc* test, such as Tukey’s test, is necessary to pinpoint specific group differences. Tukey’s test is particularly useful because it is designed to compare group means and provides confidence intervals for the differences between each pair of groups. Moreover, to gain a better understanding of the correlations among different physicochemical attributes, we employed Pearson correlation analysis to create a correlation plot using the graphing and analysis software OriginPro 2024 (OriginLab Corporation, United Staes). The graphs were created using the scientific software GraphPad Prism version 8.0.2 (San Diego, CA, United Staes) and OriginPro 2024.

## 3 Results and discussion

### 3.1 Effect of NaCl on a growth curve and morphology of *Scenedesmus* sp. BHU1

**Figure 1:**
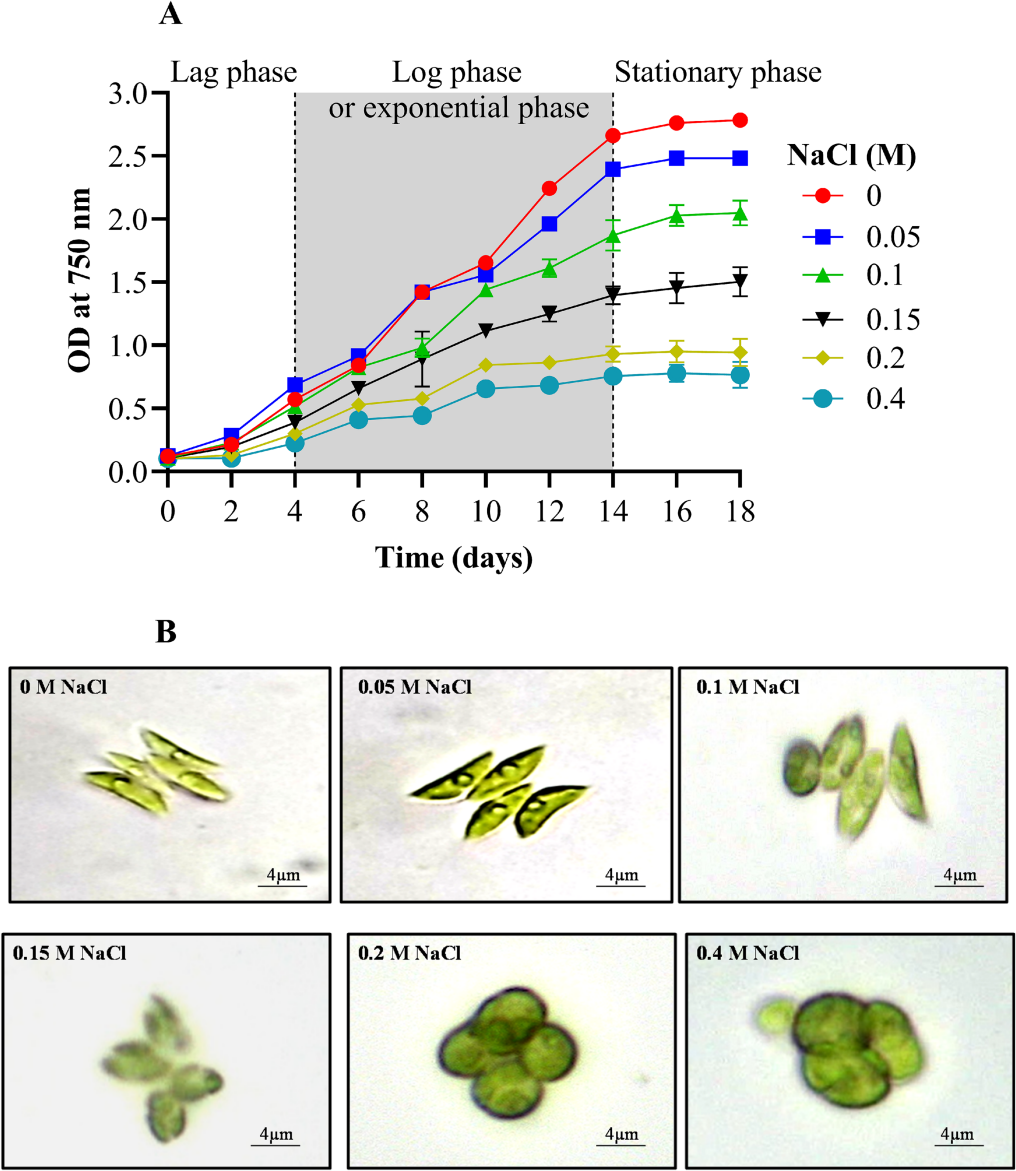
Effect of different concentrations of NaCl on growth curve **(A)** and morphology **(B)** of *Scenedesmus* sp. BHU1.

The growth of the experimental microalga *Scenedesmus* sp. BHU1 was measured turbidometrically to evaluate the effects of elevated salt on cell division. Growth was assessed at OD_750nm_, revealing that the addition of NaCl significantly (*p* < 0.05) impacted the growth of *Scenedesmus* sp. BHU1. Specifically, NaCl supplementation ranging from 0 to 0.15 M significantly accelerated the exponential growth phase between days 4 and 14 (Figure 1A). However, a decline in the growth rate was observed in cultures exposed to higher NaCl concentrations (0.2 and 0.4 M). These findings are similar to those reported by Yang et al. (2022), who documented a similar decline in the growth of freshwater microalgae *Chlorella sorokiniana* and *Desmodesmus asymmetricus* under salinity conditions of 0.3 to 0.6 M NaCl.

Furthermore, the literature underscores that while low NaCl concentrations can enhance specific metabolic pathways crucial for microalgal growth, excessive salinity levels are detrimental, leading to reduced viability and growth cessation (Fal et al., 2022). The excess salt ions disrupt the dynamic equilibrium between ROS production and consumption, ultimately causing cell death. Moreover, changes in osmotic potential and cell membrane permeability, influenced by membrane-specific ion channels, water potential, ion transport, and the solubility of CO_2_ and O_2_, contribute to shifts in cellular ionic ratios, resulting in growth inhibition (Pancha et al., 2015).

Stress conditions or changes in the environment can alter the shape or appearance of microalgae. For instance, under elevated salt content (0 to 0.4 M NaCl), the shape of *Scenedesmus* sp. BHU1 underwent a noticeable transformation, shifting from an elongated to an oval form (Figure 1B). An oval shape facilitates a more even distribution of photosynthetic pigments, such as chlorophyll, across the cell’s surface, thereby increasing its exposure to light. This alignment with incident light can be more efficient, maximizing light absorption and the utilization of available photons for photosynthesis. This shape adjustment serves as an adaptation that can help maintain or even improve photosynthetic activity under high salt stress when light energy becomes crucial for energy production (Carillo et al., 2020). We have also observed that under high salt supplementation, the *Scenedesmus* sp. BHU1 cells exhibited significant enlargement compared to the control (Figure 1B). This enlargement suggests that osmoprotectants could have accumulated within the cells as an adaptive response to high salinity (Fal et al., 2022). Their primary function is to assist cells in coping with osmotic stress by regulating water balance and safeguarding cellular structures. The enlargement of cells under increasing salt stress represents an adaptive strategy that helps the cells maintain turgor pressure, resist desiccation, and endure the saline environment. Consequently, this adaptation enables cells to counteract the adverse effects of high salt concentrations and increases their chances of survival.

### 3.2 Effect of NaCl on photosynthetic efficiency of *Scenedesmus* sp. BHU1

**Figure 2:**
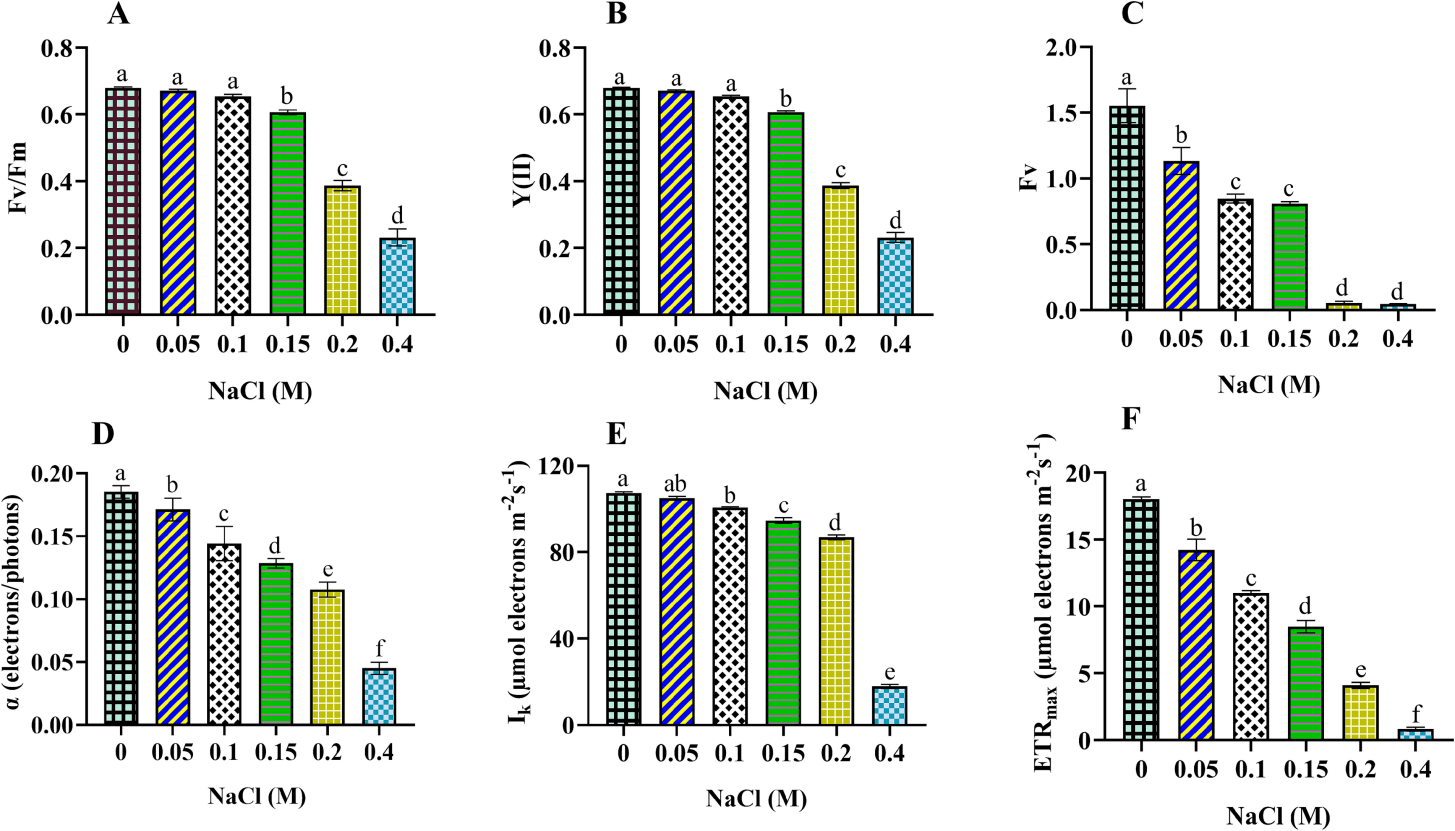
Effect of different concentrations of NaCl on photosynthetic efficiency obtained from slow kinetics and rapid light curve of PSII in *Scenedesmus* sp. BHU1. The various graph panels represent: **(A)** maximum photochemical activity (Fv/Fm), **(B)** effective photochemical activity [Y(II)], **(C)** dark-adapted variable fluorescence (Fv), **(D)** initial slope of the rapid light response curve (α in terms of electrons/photons), **(E)** minimum saturating light intensity (I_k_ in terms of μmol electrons m^−2^ s^−1^), and **(F)** maximum electron transfer rate (ETR_max_ in terms of μmol electrons m^−2^ s^−1^). The bars above the vertical columns represent the mean ± SEM of three biological replicates (*n* = 3). Different superscript lowercase alphabet letters (a–f) above the bars indicate the levels of statistically significant differences, and the values followed by the same alphabet letters refer to the statistically non-significant differences among treatment means at the *p* < 0.05 probability threshold (Tukey’s *post hoc* test).

In this section, we explored how salinity affects the photosynthetic efficiency of *Scenedesmus* sp. BHU1. The microalgal cells subjected to NaCl concentrations ranging from 0 to 0.4 M exhibited a significant (*p* < 0.05) reduction in Fv/Fm, Y(II), Fv, α, I_k_, and ETR_max_ (Figure 2). This suggests that the photosynthetic activity of *Scenedesmus* sp. BHU1 is significantly affected by high salt doses. Fv/Fm is a key indicator of maximum photosynthetic efficiency and the overall health of photosynthetic organisms. In the control cells, the Fv/Fm value was 1.8-fold higher than the value observed in cells treated with 0.4 M NaCl. A decrease in Fv/Fm signifies a reduction in the efficiency of PSII in capturing and utilizing light energy for photosynthesis (Lauritano et al., 2024). As a result, PSII is not operating at its optimal efficiency in the 0.4 M NaCl cells. This could be attributed to salt stress, which may disrupt the normal functioning of PSII, thereby reducing the health status of *Scenedesmus* sp. BHU1. This drop in Fv/Fm suggests that PSII is less effective at converting absorbed light energy into chemical energy, potentially leading to reduced photosynthetic rates and decreased growth and productivity of *Scenedesmus* sp. BHU1. Additionally, Y(II) reflects the effective photosynthetic efficiency, as it quantifies the fraction of absorbed light energy converted into chemical energy. Monitoring Y(II) can provide insights into real-time photosynthetic activity under different conditions. The Y(II) value in the control cells was 2.7 times higher than 0.4 M NaCl-treated cells. Compared to the control, the lower Y(II) value in 0.4 M NaCl cells indicates reduced PSII activity in converting absorbed light energy into chemical energy. As a result, a smaller fraction of the captured light energy is being used for photosynthesis. This is likely due to the high salt concentration impairing the electron transport chain (ETC), leading to decreased ATP and NADPH production. This implies that salt stress substantially impacts the effective photosynthetic potential of *Scenedesmus* sp. BHU1. Furthermore, in 0.4 M NaCl-treated cells, the Y(II) decrease could be associated with an increase in Y(NO). However, Y(NO) represents the passive energy loss as heat, primarily due to the closure of PSII RC. The decline in Y(II) signifies the inhibitory effect of salinity on the utilization of photochemical energy by PSII.

Additionally, variable fluorescence (Fv) measures the maximum potential efficiency of PSII. It is calculated by subtracting the minimum fluorescence (Fo) from the maximum fluorescence (Fm). Fo is the fluorescence emitted by the chlorophyll molecules when all PSII RCs are open. However, Fm is the fluorescence of chlorophyll molecules when all PSII RCs are closed. We used Fv to evaluate the health and performance of photosynthetic organisms, as deviations from the baseline Fv signal can indicate stress-induced damage to PSII or alterations in the photosynthetic apparatus. A high Fv suggests that PSII is functioning efficiently, while a low Fv indicates damaged PSII. The maximum Fv value was observed in the control cells, which were 33-fold higher than 0.4 M NaCl-treated cells. A lower value of Fv in 0.4 M NaCl-treated cells represents a smaller difference between Fo and Fm in these cells. The diminished difference between Fo and Fm indicates a significant impairment in PSII’s ability to capture and utilize light energy. This is likely since salt stress can damage PSII and other components of the photosynthetic apparatus.

Moreover, amid escalating salt stress, the utilization of RLC has been instrumental in enhancing our comprehension of the photosynthetic efficacy of *Scenedesmus* sp. BHU1. In the cells subjected to 0.4 M NaCl treatment, we observed reductions of 4.1-fold in α, 5.9-fold in I_k_, and 21.6-fold in ETR_max_ compared to the control cells. It effectively conveys that the salt-induced reductions in α, I_k_, and ETR_max_ were associated with a significant (*p* < 0.05) decrease in the light absorption of PSII. The α is associated with the effectiveness of light absorption and utilization by PSII RCs. The decrease in α value indicates that a smaller fraction of the captured light energy is used for photochemical reactions.

Moreover, I_k_ represents the light saturation point, which is a crucial parameter in studies related to photosynthesis. It represents the light exposure at which the rate of photosynthesis becomes saturated, meaning that further increases in light intensity do not lead to a proportional rise in photosynthetic activity. The cells treated with 0.4 M NaCl had a lower I_k_ value, supporting that these cells require lower light intensity for saturation. Reduced values of I_k_ in stressed *Scenedesmus* sp. BHU1 signifies an adaptive response to salt stress, allowing maximum photosynthesis at lower light intensity and potentially avoiding further damage. By reducing their I_k_ value, stressed cells are essentially conserving energy and protecting themselves from the detrimental effects of excessive light.

Additionally, ETR_max_ represents the maximum rate at which electrons are transferred through the photosynthetic ETC. In our experiment, we observed that the ETR_max_ of cells treated with 0.4 M NaCl was the lowest among all conditions. A decrease in ETR_max_ has been correlated with a lower Y(II), indicating that stressed cells are less efficient in utilizing absorbed light energy during electron transport (Gu et al., 2014). Overall, our results show that Fv/Fm, Y(II), Fv, α, I_k_, and ETR_max_ are sensitive indicators of photosynthetic efficiency in *Scenedesmus* sp. BHU1 under elevated salt content.

### 3.3 Effect of NaCl on non-regulated and regulated photochemical quenching and quenching coefficient of PSII in *Scenedesmus* sp. BHU1

**Figure 3:**
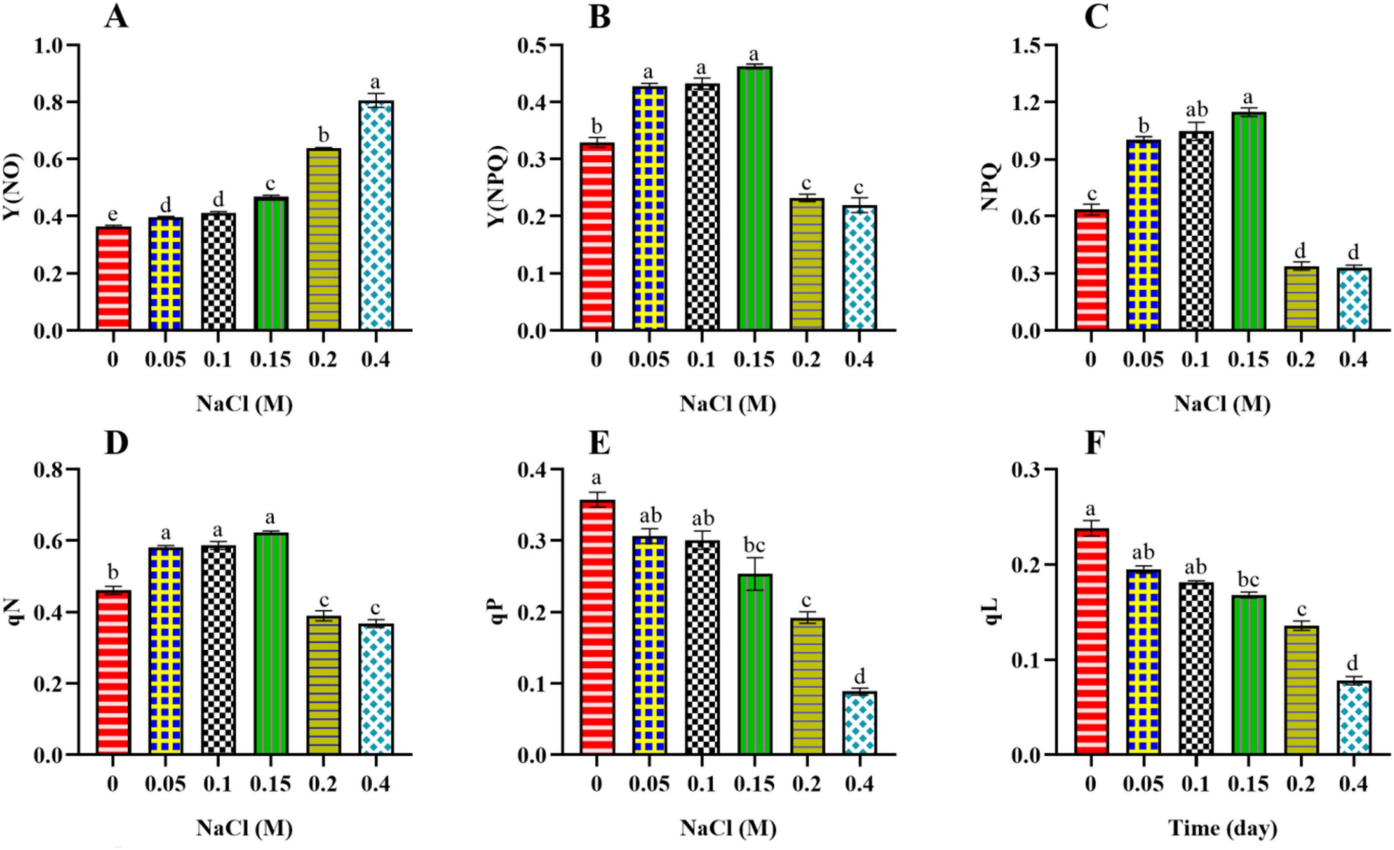
Effect of different concentrations of NaCl on non-regulated and regulated photochemical quenching [Y (NO), Y(NPQ), NPQ] and quenching coefficient (qN, qP, and qL) obtained from slow kinetics of PSII in *Scenedesmus* sp. BHU1. The various graph panels represent: **(A)** non-regulated energy dissipation [Y(NO)], **(B)** regulated energy dissipation [Y(NPQ)], **(C)** non-photochemical fluorescence quenching (NPQ), **(D)** non-photochemical fluorescence quenching coefficient (qN), **(E)** coefficient of photochemical fluorescence quenching based on the puddle model (qP), **(F)** coefficient of photochemical fluorescence quenching based on the lake model (qL), The bars above the vertical columns represent the mean ± SEM of three biological replicates (*n* = 3). Different superscript lowercase alphabet letters (a–f) above the bars indicate the levels of statistically significant differences, and the values followed by the same alphabet letters refer to the statistically non-significant differences among treatment means at the *p* < 0.05 probability threshold (Tukey’s *post hoc* test).

To delve deeper into the disruption of PSII activity, we have also assessed the distribution of absorbed, photo-excited energy between photochemical processes and heat dissipation. Non-photochemical quenching can be categorized into two types: non-regulated, represented by Y(NO), and regulated, which includes Y(NPQ) and NPQ. These components indicate the fraction of absorbed light energy that is passively dissipated as heat. The Y(NO) value increased significantly (*p* < 0.05) from control to 0.4 M NaCl-treated cells (Figure 3A). An increase in Y(NO) suggests that a larger proportion of captured light energy is being dissipated non-photochemically under elevated salt content. This increase in Y(NO) may represent an adaptive response aimed at protecting the photosynthetic machinery from excessive light energy (Liang et al., 2014). By dissipating more energy as heat, the cells may reduce the risk of photodamage to the photosystems.

In contrast, Y(NPQ) and NPQ initially increased significantly (*p* < 0.05) from control to 0.15 M NaCl-treated cells and then decreased from 0.2 to 0.4 M NaCl-treated cells (Figures 3B,C). The maximum Y(NPQ) and NPQ were found in the 0.15 M NaCl-treated cells, which were 1.5 and 1.9 times higher than control cells. The decrease in Y(NPQ) and NPQ from 0.2 to 0.4 M NaCl-treated cells indicates that at higher salt stress levels, cells are less efficient in managing excitation energy through regulated photochemical quenching. This reduced ability to quench excess energy through Y(NPQ) and NPQ could be due to stress-induced damage or limitations in the regulatory photochemical mechanisms involved (Gao et al., 2020). Furthermore, the decrease in Y(NPQ) and NPQ in cells treated with 0.2 to 0.4 M NaCl indicates a reduction in their ability to efficiently engage in non-photochemical quenching. This decrease in Y(NPQ) and NPQ further supports the idea that these cells may be experiencing challenges managing excess light energy, which could be attributed to stress-related disruptions in the regulatory photochemical mechanisms involved in non-photochemical quenching. In previous studies, the microalga *Anabaena* sp. PCC 7120 and the higher plant *Pisum sativum* showed an increase in Y(NO) and a decrease in Y(NPQ) and NPQ under cadmium and salt stress, respectively (Srivastava et al., 2021; Dhokne et al., 2022). Our findings indicate a greater loss of absorbed energy through non-regulated Y(NO) than through regulated photochemical quenching (Y(NPQ) and NPQ). This suggests that non-regulated heat dissipation plays a vital acclimatory role in protecting the photosynthetic processes of *Scenedesmus* sp. BHU1 against salt stress. Overall, our results show that the increase in Y(NO) suggests a protective mechanism to dissipate excess energy as heat, while the decrease in Y(NPQ) and NPQ may indicate impaired capacity to efficiently manage excess excitation energy, potentially due to salt-induced damage or regulatory limitations. These changes collectively reflect the cellular responses aimed at mitigating potential photodamage caused by salt stress.

Furthermore, the non-photochemical quenching coefficient (qN) quantifies the proportion of non-photochemical processes that indicate energy loss (Krause and Weis, 1991). qN represents the fraction of open PSII RCs that are actively involved in non-photochemical quenching processes. Essentially, it quantifies the proportion of PSII RCs that are closed or “quenched” to release excess excitation energy as heat. In our study, similar to the pattern observed for Y(NPQ) and NPQ, we have observed the same pattern for qN (Figure 3D). The 0.15 M NaCl-treated cells exhibited the maximum qN value, approximately 1.3 times greater than that of the control cells. The lower qN observed in 0.2 to 0.4 M NaCl-treated cells compared to 0.15 M NaCl-treated cells suggests that a smaller proportion of their PSII RCs are actively involved in NPQ processes. This reduced capacity for NPQ suggests that 0.2 and 0.4 M NaCl-supplemented cells may be unable to protect themselves from excess light energy and photodamage.

Additionally, other photochemical quenching coefficients can indicate the photochemical fraction of open reaction centers in PSII, such as qP and qL. qP suggests that absorbed light energy is distributed among multiple PSII RCs. Some of these RCs are in a quenched state (not actively participating in photochemistry), while others are open (engaged in photochemistry) (Genty et al., 1989). qL represents the fraction of open PSII RCs, indicating how many of these centers actively participate in photochemical processes and are not in a quenched state (Krause and Jahns, 2003). The values of qP and qL exhibited significant decreases (*p* < 0.05) from control cells to those supplemented with 0.4 M NaCl (Figures 3E,F). A decrease in the qP and qL indicates that a smaller fraction of PSII RCs is engaged in the photochemical processes. This reduction is often due to damage to PSII RCs caused by stress. Overall, our results indicate that PSII RCs in *Scenedesmus* sp. BHU1 were highly susceptible to salt stress, leading to a reduction in PSII photochemistry.

### 3.4 Effect of NaCl on photosynthetic parameters of fast kinetics in *Scenedesmus* sp. BHU1

#### 3.4.1 Effect of NaCl on technical fluorescence parameters of *Scenedesmus* sp. BHU1

**Figure 4:**
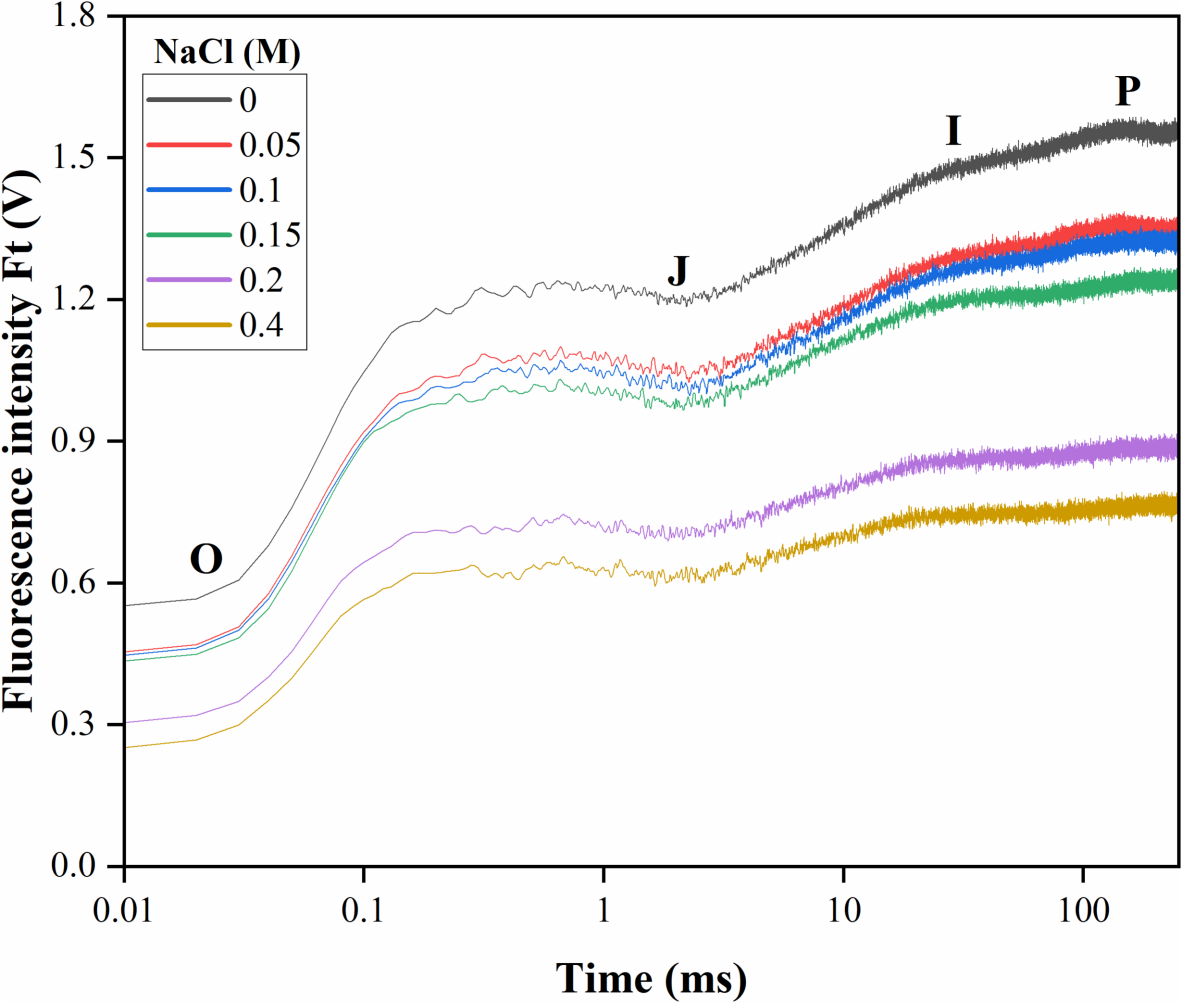
Effect of different concentrations of salt on the OJIP transient curve of *Scenedesmus* sp. BHU1 plotted on a logarithmic time scale. Fluorescence values are expressed as Ft (V), which represents measured fluorescence intensity in each time interval. The curve is drawn using the average of three biological measurements. The characteristic levels of fluorescence increase kinetics are denoted by the letters O, J, I, and P.

Elevated salt exposure significantly affected the OJIP transient curve of *Scenedesmus* sp. BHU1 provides insights into the electron transport from PSII to PSI through Q_A_ and Q_B_ (Figure 4). We noted that after salt exposure, fluorescence levels were significantly decreased (*p* < 0.05) at levels O (F_O_), J (F_J_), I (F_I_), and P (F_M_) phases in cells treated with 0 to 0.4 M NaCl (Table 1). The initial ChlF level O (F_O_) represents the minimum fluorescence emission after dark adaptation when all Q_A_ molecules are oxidized. The level J (F_J_) represents the fluorescence intensity at 2 ms. It indicates the fluorescence emission from the dark-adapted state (F_O_) to the light-adapted state. The J point provides insights into the efficacy of energy transfer within PSII and the reduction of Q_A_. Level I (F_I_) indicates the fluorescence emission at 30 ms and reflects the high fluorescence emission during the light-adapted state, specifically when Q_A_ is reduced. This level indicates the efficacy of electron transport from Q_A_ to subsequent components of the ETC. The level P (F_M_) is the maximum fluorescence emission when all Q_A_ molecules are reduced. In this state, Q_A_ accepts electrons, and there is a high level of electron flow within PSII.

**Table 1:** Basic fluorescence parameters were extracted from the OJIP transient curve.

	Basic fluorescence parameters extracted from the OJIP transient curve				
NaCl (M)	F_O_	F_K_	F_J_	F_I_	F_M_
0	0.587 ± 0.002^a^	1.247 ± 0.001^a^	1.207 ± 0.003^a^	1.498 ± 0.001^a^	1.594 ± 0.001^a^
0.05	0.488 ± 0.002^b^	1.130 ± 0.002^b^	1.063 ± 0.001^b^	1.302 ± 0.001^b^	1.388 ± 0.001^b^
0.1	0.470 ± 0.002^c^	1.085 ± 0.001^c^	1.025 ± 0.001^c^	1.282 ± 0.002^c^	1.345 ± 0.002^c^
0.15	0.457 ± 0.001^d^	1.024 ± 0.001^d^	0.996 ± 0.001^d^	1.224 ± 0.001^d^	1.263 ± 0.002^d^
0.2	0.334 ± 0.002^e^	0.757 ± 0.001^e^	0.721 ± 0.001^e^	0.884 ± 0.001^e^	0.917 ± 0.001^e^
0.4	0.286 ± 0.002^f^	0.677 ± 0.001^f^	0.635 ± 0.002^f^	0.765 ± 0.001^f^	0.781 ± 0.006^f^

The data is the mean ± SEM of three biological replicates (*n* = 3). Different superscript lowercase alphabet letters (a–f) above the values indicate the levels of statistically significant differences, and the values followed by the same alphabet letters refer to the statistically non-significant differences among treatment means at the *p* < 0.05 probability threshold (Tukey’s *post hoc* test). The meaning of these parameters is as follows: minimum ChlF intensity at 20 μs (F_O_), ChlF intensity at 0.3 ms (F_K_), ChlF intensity at 2 ms (F_J_), ChlF intensity at 30 ms (F_I_), and maximum ChlF intensity (F_M_).

Consequently, the maximal number of chlorophyll molecules excites, leading to the highest possible fluorescence signal. Changes in F_M_ levels typically reflect thermal dissipative events in PSII (Maxwell and Johnson, 2000). The decreased F_M_ in cells treated with elevated salt indicates that PSII is not actively involved in thermally dissipative events and energy quenching. This decrease in F_M_ is a sign of impaired PSII functioning, which reduces photosynthetic efficiency. Additionally, elevated salt stress also caused a decrease in fluorescence emission at levels J (F_J_) and I (F_I_). Deviations from the typical patterns observed during these phases may indicate stress-induced damage, altered electron transport rates, or changes in the electron donor and acceptor pools of PSII.

#### 3.4.2 Effect of NaCl on energy flux per reaction center and per cross-section of PSII in *Scenedesmus* sp. BHU1

**Figure 5:**
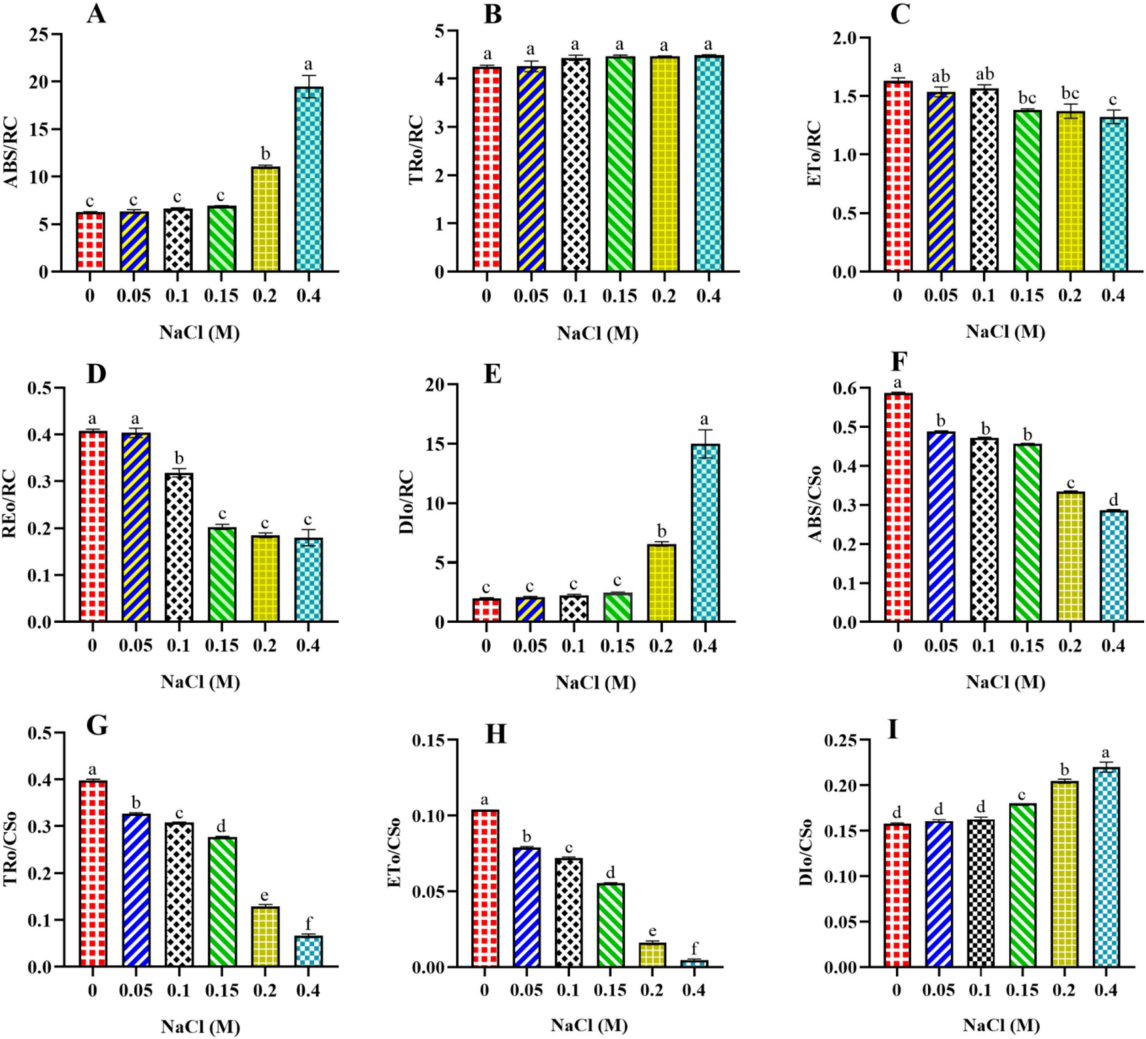
Effect of different concentrations of NaCl on specific energy fluxes per reaction center and per cross-section of PSII in *Scenedesmus* sp. BHU1. The different panels represent: **(A)** absorption energy flux per active reaction center (ABS/RC), **(B)** trapping energy flux per active reaction center (TR_O_/RC), **(C)** electron transport flux per active reaction center (ET_O_/RC), **(D)** electron flux reducing end electron acceptors at the PSI acceptor side per RC (REo/RC), **(E)** dissipation of excess energy per active reaction center (DI_O_/RC), **(F)** absorption energy flux per excited sample cross-section (ABS/CS_O_), **(G)** trapping energy flux per excited sample cross-section (TR_O_/CS_O_), **(H)** electron transport flux per excited sample cross-section (ET_O_/CS_O_), **(I)** dissipation of excess energy per excited sample cross-section (DI_O_/CS_O_). The bars above the vertical columns represent the mean ± SEM of three biological replicates (*n* = 3). Different superscript lowercase alphabet letters (a–f) above the bars indicate the levels of statistically significant differences, and the values followed by the same alphabet letters refer to the statistically non-significant differences among treatment means at the *p* < 0.05 probability threshold (Tukey’s *post hoc* test).

The study used fast kinetic fluorescence transient responses to understand the energy transfer mechanisms from PSII to PSI. These functional parameters were determined using specific energy fluxes per active reaction center (RC), energy fluxes per excited cross-section (CS), flux ratios (quantum efficiencies), and performance index. This section discusses the specific energy fluxes per active RC and per excited CS of PSII in *Scenedesmus* sp. BHU1. Furthermore, information about the transmission of absorbed photon energy was derived from factors including ABS/RC, TRo/RC, ETo/RC, REo/RC, DIo/RC, ABS/CSo, TRo/CSo, ETo/CSo, and RC/CSo. These factors have been calculated using basic fluorescence parameters extracted from the OJIP transient curve, as provided in Table 1. ABS/RC is a marker of actual antenna size (gross chlorophyll per functional RC), and an improvement in this factor indicates that the antenna’s apparent size has increased. The ABS/RC ratio significantly increased (*p* < 0.05) from control cells to those treated with 0.4 M NaCl, while the TRo/RC ratio increased non-significantly (*p* > 0.05) (Figures 5A,B). The maximum values of ABS/RC and TRo/RC were observed in the 0.4 M NaCl-treated cells, being 3.1-fold and 1-fold greater than those in the control cells, respectively. This suggests that the increase in active PSII RCs has led to a greater amount of photon energy being absorbed and trapped per active RC. Under salt stress, it is possible that *Scenedesmus* sp. BHU1 activates additional PSII RCs to capture more photon energy. This activation of extra RCs may serve as an adaptive mechanism to compensate for the reduced photosynthetic efficiency caused by salt stress. Furthermore, high salt stress conditions can change the pigment composition within *Scenedesmus* sp. BHU1. These changes in pigments can directly affect the absorption capacity of PSII RCs. Therefore, the observed increase in ABS/RC may be attributed to this stress-induced alteration in pigment composition, which enables *Scenedesmus* sp. BHU1 to maximize its photon energy capture and maintain its photosynthetic performance. Furthermore, the significant decrease (*p* < 0.05) in ETo/RC and REo/RC from 0 to 0.4 M NaCl-treated cells indicates a specific impairment in the competency of PSII to transform trapped emission energy into electron transport (Figures 5C,D). The maximum values of ETo/RC and REo/RC were observed in the control cells, which were 1.2-fold and 2.2-fold higher than those in the 0.4 M NaCl-supplemented cells. This suggests that more energy is being re-emitted as fluorescence rather than being used for electron transport (Huang et al., 2016). Contrary to this, DIo/RC significantly increased (*p* < 0.05) from control cells to those treated with 0.4 M NaCl (Figure 5E). This increase was 7.4-fold higher in the 0.4 M NaCl-treated cells as compared to the control cells, serving to shield the photosynthetic machinery from photodamage. The increase in DI_O_/RC may be associated with the activation of energy-quenching mechanisms, such as NPQ. NPQ helps dissipate excess energy as heat and involves changes in the conformation of the light-harvesting complexes (LHCs), to reduce their ability to absorb light. Excess energy dissipation serves as a protective mechanism because it prevents the over-excitation of PSII and minimizes the production of harmful ROS, which can damage the photosynthetic machinery and other cellular components. Canora et al. (2022) have shown that the algae *Klebsormidium* sp. and *Stigeoclonium* sp. had higher DI_O_/RC values under 0.3 M NaCl. These results indicate that *Scenedesmus* sp. BHU1 exhibited acclimatization responses to higher salt content, suggesting its ability to adapt and cope with challenging environmental conditions.

Furthermore, the present study showed that the ABS/CS_O_, TR_O_/CS_O_, and ET_O_/CS_O_ values significantly decreased (*p* < 0.05) from control cells to those treated with 0.4 M NaCl (Figures 5F–H). The maximum ABS/CSo, TRo/CSo, and ETo/CSo were found in the control cells, which were 2-fold, 10.5-fold, and 20-fold higher than those in the cells treated with 0.4 M NaCl, respectively. This result suggests that increasing salt concentrations may lead to alterations in the ultrastructure of chloroplasts, where PSII is located. Changes in chloroplast morphology can affect the arrangement and function of pigment molecules, potentially decreasing the ABS/CSo value. Another reason could be that increasing salinity stress is known to reduce the concentration of photosynthetic pigments. A decrease in pigment concentration directly impacts the ability of PSII to absorb light energy. The significant decrease (*p* < 0.05) in TR_O_/CS_O_ suggests that, under increased salt content, the efficiency of converting absorbed light energy into useful energy for photosynthesis has been decreased. This could be due to the disruptions in the photosynthetic ETC. The significant decrease (*p* < 0.05) in ETo/CSo suggests that less absorbed energy is emitted during electron transport per cross-section. It is possible that the decrease in TRo/CSo and ETo/CSo is an acclimation response to elevated salt content. The cells might redirect energy into protective mechanisms or metabolic pathways that help them survive stressful conditions. This suggests that salt-treated cells of *Scenedesmus* sp. BHU1 exhibited a reduced capacity for PSII to transform emission energy into electron transport, resulting in a significant increase (*p* < 0.05) in the DI_O_/CS_O_ value (Figure 5I). The increase in DIo/CSo suggests that stressed cells dissipate excess energy as heat. A similar outcome was observed in *Anabaena* PCC 7120 under cadmium stress, where PSII proteins (CP43, CP47, D1, and D2) were severely inhibited (Srivastava et al., 2021). The CP43 and CP47 proteins are specific light-harvesting proteins that transfer energy collected from the peripheral LHCs to the PSII core proteins D1 and D2 (Roose et al., 2007). Hence, the considerable decline in the concentration of all four PSII proteins (CP43, CP47, D1, and D2) may result in ineffective photon absorption and reduced PSII electron transfer activity by adversely impacting the structural organization of PSII. When microalgae are exposed to salt stress, photosynthetic cells regularly produce ROS that particularly target the LHCs and PSII RC core D1 proteins (Kaur et al., 2022). Photosynthesis is regulated by a sophisticated repair process that involves the removal of the damaged D1 core protein and the synthesis of a new protein copy to integrate into the PSII RCs. The cellular redox state carefully governs the multi-step process of forming and incorporating the new D1 protein copy. These responses show that *Scenedesmus* sp. BHU1 can acclimatize to high salt stress by prioritizing photoprotection over maximal photosynthetic efficiency.

#### 3.4.3 Effect of NaCl on energy flux ratio, density, and performance index of PSII in *Scenedesmus* sp. BHU1

**Figure 6:**
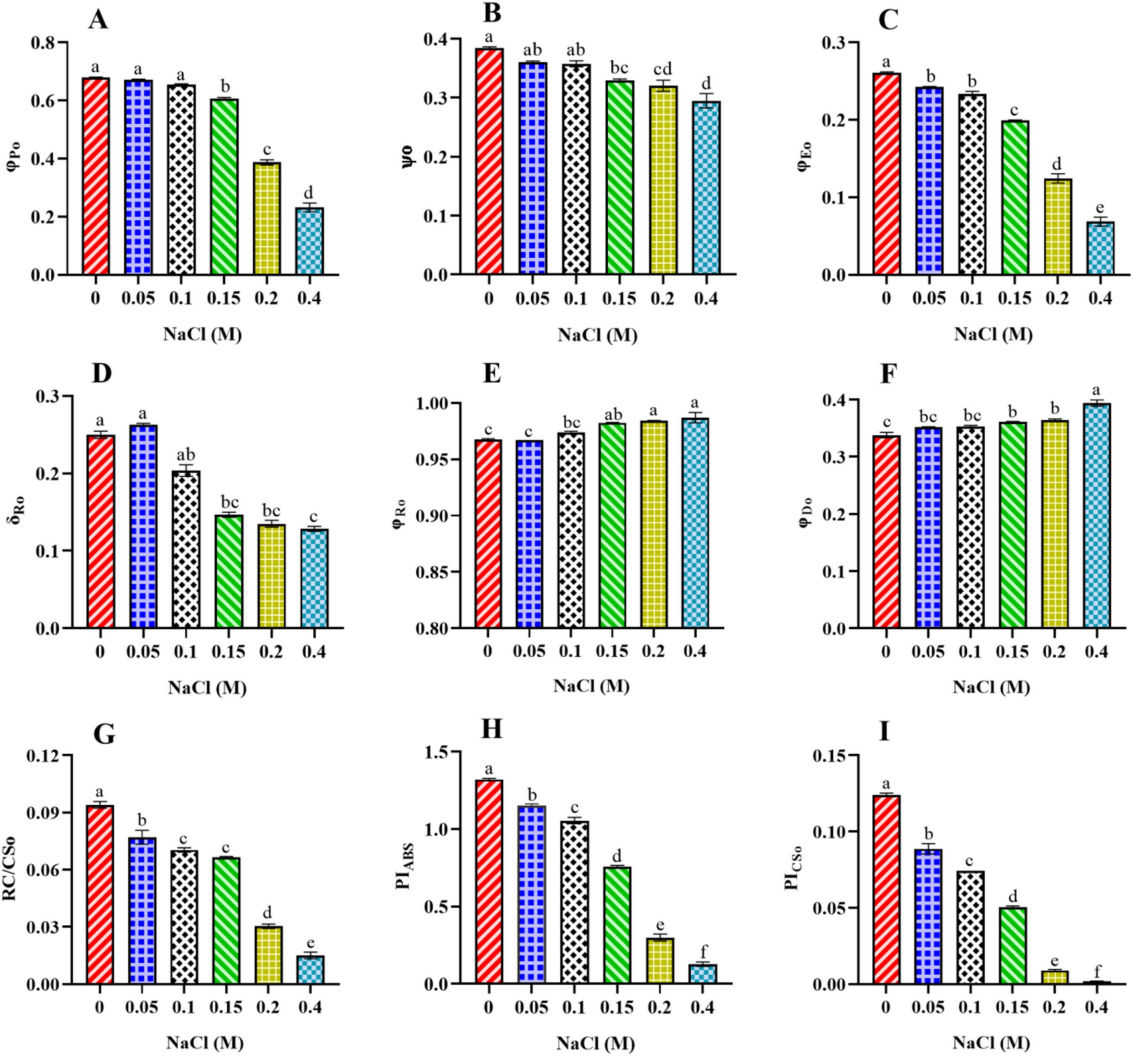
Effect of different concentrations of NaCl on energy flux ratio, density, and performance index of PSII in *Scenedesmus* sp. BHU1. The different panels represent: **(A)** photosynthetic integrity of primary PSII photochemistry (φ_Po_), **(B)** efficiency/probability with which a PSII-trapped electron is transferred further than Q_A_− into the ETC (ѱo), **(C)** quantum yield for electron transport from Q_A_− to PQ (φ_Eo_), **(D)** efficiency/probability with which an electron is transferred from Q_B_ to PSI acceptors (δ_Ro_), **(E)** quantum yield for reduction of end electron acceptors at the PSI acceptor side (φ_Ro_), **(F)** quantum yield of energy dissipation (φ_Do_), **(G)** density of RCs (Q_A_− reducing RC per CS of PSII) at t = t_FM_ (RC/CSo), **(H)** performance index (potential) for energy conservation from photons absorbed by PSII to the reduction of intersystem electron acceptors (PI_ABS_), **(I)** performance index based on cross-section at t = t_FM_ (PI_CSo_). The bars above the vertical columns represent the mean ± SEM of three biological replicates (*n* = 3). Different superscript lowercase alphabet letters (a–f) above the bars indicate the levels of statistically significant differences, and the values followed by the same alphabet letters refer to the statistically non-significant differences among treatment means at the *p* < 0.05 probability threshold (Tukey’s *post hoc* test).

Additional findings revealed that φ_Po_, which represents the F_V_/F_M_ parameter and measures the physiological status of a photosynthetic organism, significantly decreased from control cells to those treated with 0.4 M NaCl (Figure 6A). The decrease in φ_Po_ may indicate a reduction in electron transport efficiency within PSII. Stress can lead to the disruption of the ETC, resulting in a decline in the conversion of absorbed light energy into chemical energy (Roose et al., 2007). Also, the significant decrease (*p* < 0.05) in the values of Ѱo from control to 0.4 M NaCl-treated cells suggests that PSII-trapped electrons do not properly enter the ETC beyond Q_A_− in salt-treated cells (Figure 6B). The efficiency of electron transfer beyond Q_A_− has been influenced by the redox state of Q_A_− itself. If Q_A_− is efficiently reduced (accepting electrons), it allows for the continuation of electron flow in the ETC. Under increasing salt concentration, QB’s electron acceptance redox potential may be more negative than Q_A_−, which may explain the decreased value of ѱ_O_ at higher salt content. Another possible reason is that, under enhanced salt content, cells may not activate alternative electron acceptors downstream of Q_A_− or electron sinks to reduce the risk of overexcitation in the photosystem.

**Figure 7:**
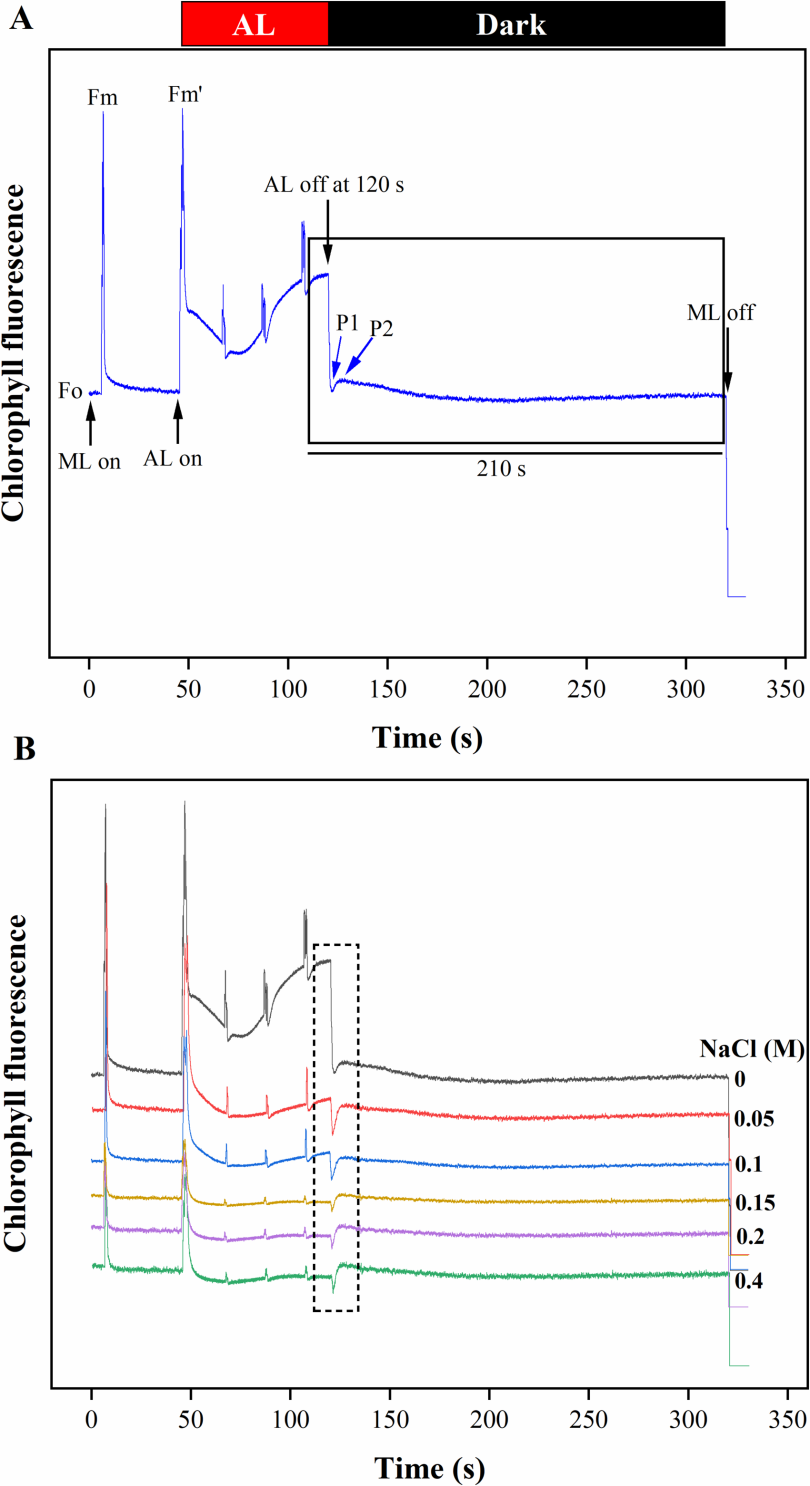
The effect of salt stress on the amplitude of the post-illumination transient rise in ChlF was measured to assess CEF around PSI in *Scenedesmus* sp. BHU1. The graph panels show **(A)** the curve, which shows a typical trace of ChlF in the control sample. The transient rise in ChlF that occurs after switching off AL (104 μmol photons m^−2^ s^−1^, at 120 s) with ML background is highlighted by the black box. The small up and down arrows (black color) indicate turning on and off ML and AL, respectively. F_O_ is a dark-adopted minimal fluorescence yield, F_M_ is a dark-adopted maximal fluorescence yield after activating the saturation pulse, F_M_′ is a light-illuminated maximal fluorescence yield after activating the AL, peak 1 (P1) represents the fast phase of PIFT generated by NADPH and/or Fd and peak 2 (P2) represents the slow phase of PIFT generated by the formation of glucose and triose phosphate. **(B)** Increased transition from LEF to CEF from control to 0.4 M NaCl treated cells.

Furthermore, the significant drop in the φ_Eo_ value from control to 0.4 M NaCl-treated cells suggests that the PSII electron transfer from Q_A_− to PQ was slowed down (Figure 6C). The reduction is likely a result of salt stress-induced impairments, photoinhibition, protective mechanisms, or acclimation responses that collectively affect the functionality of the electron transport chain. It highlights *Scenedesmus* sp. BHU1 responds to stress by modulating its photosynthetic processes to mitigate potential damage and maintain cellular integrity. Additionally, we observed a significant decrease (*p* < 0.05) in the δ_Ro_ value from control to 0.4 M NaCl-treated cells, while the φ_Ro_ value showed a significant increase (*p* < 0.05) within the same concentration range (Figures 6D,E). The significant decrease in δ_Ro_ suggests that, under elevated salt stress, there has been a reduction in the efficiency of electron transfer from Q_B_ to PSI acceptors. The significant increase in φ_Ro_ implies that the quantum yield for the reduction of end electron acceptors at the PSI acceptor side may have increased in high salt-treated cells. This suggests that there might have been a subtle increase in electron transport efficiency on the PSI acceptor side in salt-treated cells. The increased φ_Ro_ is crucial for optimizing photosynthetic efficiency while protecting against photodamage. Our findings imply that electron transfer efficiency from the cyt b_6_f complex to PSI acceptors has increased, which could be associated with PSI-driven CEF induction and the formation of ATP via cyclic photophosphorylation. This hypothesis was confirmed by an increased post-illumination transient in ChlF amplitude, which indicated that NDH-1-based CEF around PSI was activated in salt-treated cells (Figure 7). The increase in the quantum yield of energy dissipation (φ_Do_) from control to 0.4 M NaCl-treated cells can be attributed to the microalgae response to stress (Figure 6F). This increased φ_Do_ signifies a more efficient dissipation of excess energy as heat, reducing the risk of ROS production and subsequent cellular damage.

The decrease in the functional density RC/CSo (Q_A_− reducing RC per CS of PSII) from control to 0.4 M NaCl-treated cells could be attributed to various factors affecting the PSII complex (Figure 6G). This damage is due to the over-absorption of light energy, which can harm the RCs per CS of PSII. This results in a decrease in the density of functional RCs, potentially affecting their ability to effectively participate in the ETC. Further, increasing salt concentrations can impact the synthesis of PSII proteins, including those that constitute the RCs. Reduced synthesis or accelerated degradation of these proteins may contribute to a decrease in the density of functional RCs per CS of PSII. The performance index (potential) for energy conservation, as measured by per RC (PI_ABS_) and per CS (PI_CSo_), both indicate the efficiency of electron flow from PSII to the PQ pool (Srivastava et al., 1999). The values of both PI_ABS_ and PI_CSo_ significantly decreased (*p* < 0.05) from control to 0.4 M NaCl-treated cells (Figures 6H,I). The maximum values of PI_ABS_ and PI_CSo_ were observed in the control cell, which were 10- and 62-fold higher, respectively, than the 0.4 M NaCl-treated cells. This may indicate a disruption in the electron flow from PSII RC to the PQ pool. Such disruption could be caused by alterations in the protein complexes involved in the ETC or changes in the redox potential of the components. Any impairment in PSII functionality can disrupt the flow of electrons. Another reason could be the disruption caused by damage to electron carriers or other components of the chain. Changes in PQ pool dynamics can influence the overall performance index. Additionally, under elevated salt stress, algae may activate protective mechanisms, such as NPQ, to dissipate excess light energy and prevent damage. While these mechanisms are essential for survival, they can also lead to a decrease in the measured performance index for energy conservation (Sukhova and Sukhov, 2018). As a result, electrons may not flow smoothly from PSII to the interstitial electron acceptors, leading to a decrease in the efficiency of electron transfer.

### 3.5 Effect of NaCl on post-illumination chlorophyll fluorescence transient in *Scenedesmus* sp. BHU1

The CEF activity around PSI, mediated by NDH-1, has been studied using PIFT in microalgae (Xu et al., 2016; Srivastava et al., 2022). The abiotic stress-related photoacclimatory response involves the induction of PSI-driven CEF, which is measured as PIFT (Shikanai et al., 1998). The fast phase, peak 1 (P1), which is one of the two peaks in PIFT, is generated by NADPH and/or Fd, while the slow phase, peak 2 (P2), is generated by respiratory substrates such as glucose and triose phosphate (Xu et al., 2016; Srivastava et al., 2022). After 120 s of switching off AL, the control cells showed lower amplitudes at the P1 and P2 peaks, while cells treated with increasing concentrations of NaCl (ranging from 0.05 to 0.4 M) demonstrated higher amplitudes at the P1 and P2 peaks (Figure 7B). This finding suggests that the fast phase of the kinetics represents the rapid CEF around PSI, which donates electrons to the PQ pool from the reduced Fd in PSI. The amplitude at the P2 peak was higher than at the P1 peak in both control and salt-treated cells (Figure 7B). However, a greater increase in the amplitude of peak P2 was observed in salt-treated cells, resulting in an increased amount of electrons from photoreductants. According to Srivastava et al. (2022), PSI-dependent CEF increases during abiotic stress due to the improved stability of the ATP synthase enzyme and the Cyt b_6_f complex. Several CEF modes have been proposed for microalgae, but the mechanism with the highest quantum yield involves moving electrons from NADPH to the PQ pool via NDH-1. Microalgae have been shown to contain several NDH-1 isoforms, including NDH-1MS, NDH-1MS, NDH-1 L, and NDH-1 L’, all of which are involved in CEF (Zhang et al., 2020). PSI and NDH-1 combine to form the supercomplex PSI-NDH-1, which is essential for surviving various abiotic stressors by participating in CEF. Recently, it has been suggested that, under stress, PSII activity decreases more than PSI activity in microalgae (Srivastava et al., 2022). According to our findings, the increase in both the fast and slow peaks in stressed cells indicates a strong link between CO_2_ assimilation activity and CEF. Xu et al. (2016) reported that CEF around PSI is crucial for producing ATP for carbon assimilation.

### 3.6 Effect of NaCl on the viability of *Scenedesmus* sp. BHU1

This section explores fluorescence-based assays to assess cell viability during salt treatments using fluorescein diacetate (FDA) dye. FDA is a non-fluorescent compound that penetrates intact cell membranes. Inside viable cells, FDA is converted by cellular esterases into fluorescein, a green fluorescent compound (Antoniadi et al., 2022). Therefore, green fluorescence indicates viable cells. This conversion occurs in metabolically active cells with intact membranes. A decrease in cell viability, indicated by reduced green fluorescence, suggests that increasing concentrations of NaCl (ranging from 0.05 to 0.4 M) are affecting the metabolic activity and/or membrane integrity of *Scenedesmus* sp. BHU1 cells (Supplementary Figure S1). This could be due to several reasons: Elevated salt concentrations may impair cellular metabolism, leading to a decrease in the activity of esterases responsible for FDA conversion. As a result, fewer cells exhibit green fluorescence in the 0.4 M NaCl-treated group. Increasing salt content in the cells can also damage cell membranes, compromising their integrity. This can lead to leakage of FDA and other cellular components, reducing the availability of FDA for conversion to fluorescein in the 0.4 M NaCl-treated cells.

### 3.7 Effect of NaCl on stress biomarkers, osmoprotectants, and intracellular cations of *Scenedesmus* sp. BHU1

**Figure 8:**
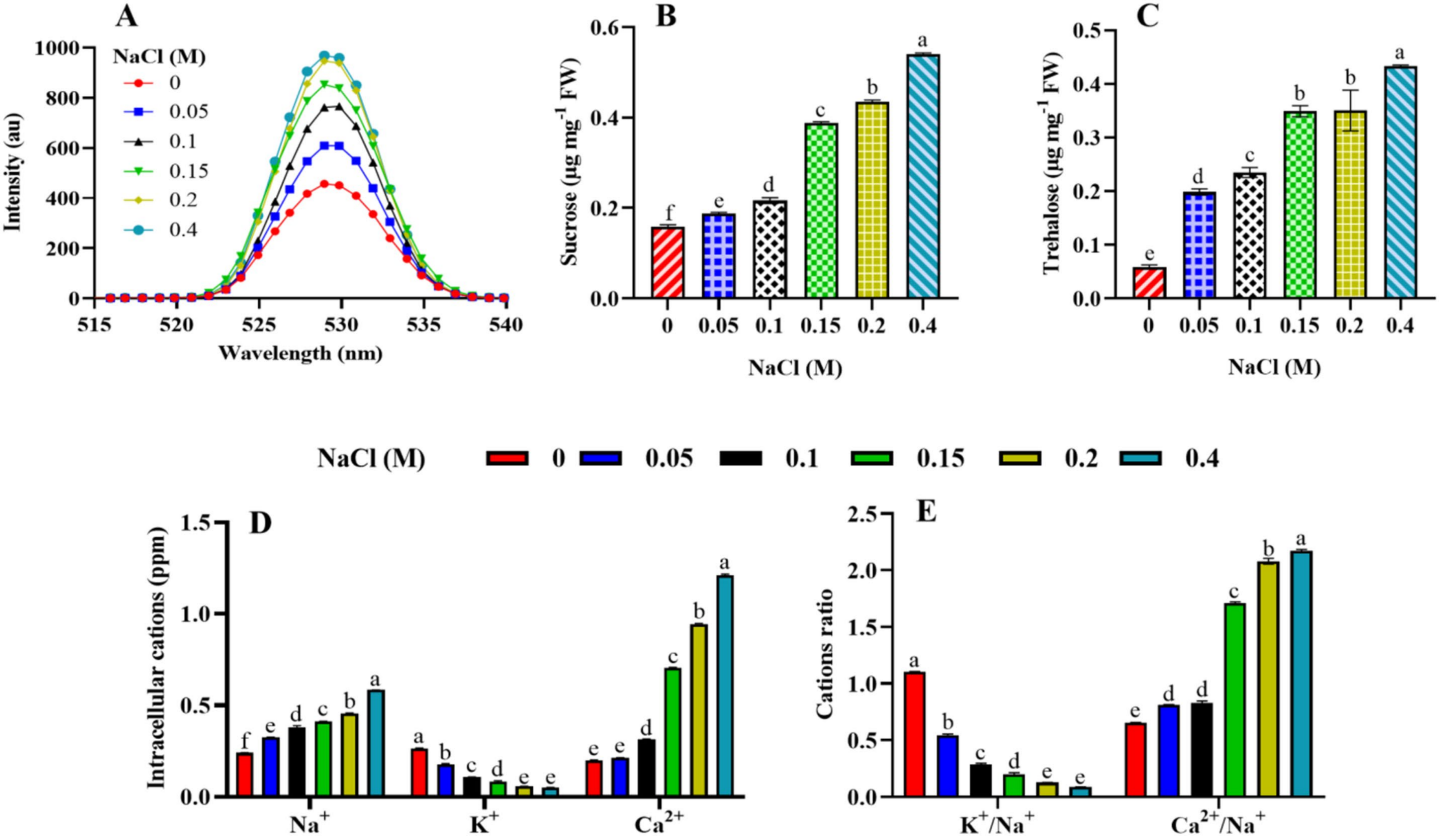
Effect of different concentrations of NaCl on the stress biomarker, osmoprotectants, and intracellular cations of *Scenedesmus* sp. BHU1. The different panels represent **(A)** stress biomarkers: reactive oxygen species (ROS), **(B,C)** osmoprotectants (sucrose and trehalose), and **(D,E)** intracellular cations (cations and cation ratio). The bars above the vertical columns represent the mean ± SEM of three biological replicates (*n* = 3). Different superscript lowercase alphabet letters (a–f) above the bars indicate the levels of statistically significant differences, and the values followed by the same alphabet letters refer to the statistically non-significant differences among treatment means at the *p* < 0.05 probability threshold (Tukey’s *post hoc* test).

In the present study, the effect of elevated salt content on ROS production was assessed by monitoring the fluorescence level of DCF dye. The results reveal that an increase in NaCl content caused a significant increase in ROS accumulation in *Scenedesmus* sp. BHU1. The concentration of ROS increased from the control to the 0.4 M NaCl-treated cells, suggesting a stress response in this organism (Figure 8A). Similar observations were also reported in the green microalga *C. reinhardtii* under 0.2 M NaCl grown culture (Fal et al., 2022). Higher concentrations of NaCl impose osmotic stress on cells. *Scenedesmus* sp., being a freshwater microalga, might not be well adapted to high salt concentrations. To counteract this stress, cells may activate mechanisms that generate ROS. ROS, such as superoxide anion radical (O_2_^•−^), hydrogen peroxide (H_2_O_2_), and hydroxyl radical (HO^•^), often serve as signaling molecules in stress responses. They can trigger various protective mechanisms to cope with stress conditions, such as activating antioxidant enzymes and increasing the production of osmoprotectants. While ROS can play beneficial roles as signaling molecules, excessive ROS accumulation can lead to cellular damage. This includes oxidative damage to lipids, proteins, and nucleic acids, decreased nutrient uptake, reduced CO_2_ flux, and inducing thylakoid membrane lipid peroxidation (Pancha et al., 2015; Fal et al., 2022). Photosynthetic organisms have evolved osmoprotectants and antioxidant defense systems to mitigate ROS-induced damage. These include osmoprotectants (proline, sucrose, and trehalose), enzymatic antioxidants (SOD, CAT, APX, and POD), and non-enzymatic antioxidants (glutathione and ascorbate). An increase in ROS production might trigger the activation of these defense mechanisms. The response of *Scenedesmus* sp. BHU1 to increasing NaCl concentrations can vary depending on the severity and duration of exposure. At lower concentrations, the increase in ROS production may be part of an adaptive response aimed at maintaining cellular homeostasis. However, at higher concentrations, ROS accumulation could exceed the capacity of antioxidant defense systems, leading to toxicity and cell death.

Osmoprotectant molecules are crucial components of the defense mechanism in microalgae and become highly activated in response to stress. The current study examined sucrose and trehalose for their roles as osmoprotectants. Under elevated salt stress, the levels of sucrose and trehalose in plants and microalgae can be affected as part of their response mechanisms to cope with the stress. In our case, the microalga *Scenedesmus* sp. BHU1 accumulates the highest levels of sucrose in 0.4 M NaCl-treated cells, which are 3.3 times higher than those in control cells (Figure 8B). As the salt concentration rises, cells may experience osmotic stress due to the higher external solute concentration. In response to this stress, the microalga might synthesize and accumulate sucrose as an osmolyte. Sucrose acts as an osmoprotectant by helping cells maintain their turgor pressure and prevent dehydration in a hypertonic environment. This adaptive response allows the microalga to thrive and survive under conditions of elevated salt content. Furthermore, we found that the maximum level of trehalose in 0.4 M NaCl-treated cells was 7.3-fold higher than in control-grown cells (Figure 8C). This is because trehalose helps maintain cellular turgor by acting as an osmoprotectant. As increasing salt stress causes water to move out of the microalgal cells, the accumulation of trehalose aids in retaining water within the cells and prevents dehydration (Swapnil and Rai, 2018). Moreover, trehalose has protective properties against protein denaturation and membrane damage. Under increasing salt concentrations, there is a greater risk of protein and membrane destabilization. Trehalose helps stabilize these structures, maintaining the integrity and functionality of cellular components. Additionally, increasing salt stress can lead to the generation of ROS in microalgal cells, causing oxidative stress. Trehalose has antioxidant properties and can help scavenge ROS, reducing oxidative damage and enhancing the microalgae’s ability to cope with stress. Furthermore, trehalose has been suggested to function as a signaling molecule in stress response pathways. Its accumulation may trigger specific stress-related gene expression and metabolic adjustments, contributing to the overall stress tolerance of the microalgae. Finally, trehalose metabolism is also interconnected with other metabolic pathways. Its accumulation can influence the balance between sugar metabolism and energy production, facilitating the microalga’s adaptation to altered conditions induced by higher salt levels. Therefore, the dose-dependent increase in the concentration of these osmoprotectants in *Scenedesmus* sp. BHU1 indicates a well-coordinated adaptive response to elevated salinity stress.

The intracellular Na^+^, K^+^, and Ca^2+^ levels, as well as the K^+^/Na^+^ and Ca^2+^/Na^+^ ratios in the microalgal cells, were considerably affected by increasing salt concentrations. Enhanced salinity in the growing medium significantly (*p* < 0.05) increased Na^+^ and Ca^2+^ levels, as well as the Ca^2+/^Na^+^ ratio, while decreasing K^+^ levels and the K^+^/Na^+^ ratio in *Scenedesmus* sp. BHU1. The Na^+^ and Ca^2+^ levels, as well as the Ca^2+^/Na^+^ ratios, were highest in 0.4 M NaCl-treated cells (Figures 8D,E). This indicates the physiological response of the microalgae to maintain cellular homeostasis in the presence of high salt concentrations. Accordingly, a prior study found that salt stress considerably increased Na^+^ and Ca^2+^ cations and Ca^2+^/Na^+^ ratios in *Anabaena fertilissima* (Swapnil and Rai, 2018). The observed increase in Na^+^ and Ca^2+^ levels and the decrease in K^+^ levels in *Scenedesmus* sp. BHU1 under 0.4 M NaCl treatment indicate a significant disruption in ion homeostasis. The high salt content in the surrounding environment often leads to an influx of Na^+^ into the cells due to osmotic stress. This influx results from the imbalance between external and internal Na^+^ concentrations, driving Na^+^ uptake (Pancha et al., 2015). One of the primary reasons for the increased Na^+^ levels is to counteract the osmotic stress caused by high salt concentrations in the culture. By accumulating Na^+^, *Scenedesmus* sp. BHU1 can balance the osmotic pressure inside and outside the cell, preventing excessive water loss and maintaining cell turgor. However, while Na^+^ accumulation helps in osmoregulation, excessive levels of Na^+^ can be toxic to *Scenedesmus* sp. BHU1 cells. High concentrations of Na^+^ can disrupt cellular processes and lead to cellular damage or even cell death. Furthermore, Ca^2+^ plays an essential role in maintaining cell structure and integrity. Under increasing salt concentrations, *Scenedesmus* sp. BHU1 may increase the uptake of Ca^2+^ to reinforce cell walls and membranes, providing structural support against the adverse effects of high salinity. Ca^2+^ also acts as a secondary messenger in various cellular signaling pathways. The increase in Ca^2+^ levels may trigger specific signaling cascades involved in the response to salt stress, helping *Scenedesmus* sp. BHU1 adapt to the changing culture medium. The increase in Na^+^ and Ca^2+^ levels reflects the microalgae’s efforts to maintain ion homeostasis under elevated salt stress (Swapnil and Rai, 2018). These ions play crucial roles in balancing osmotic pressure, supporting cellular structures, and regulating cellular signaling pathways. The decrease in K^+^ levels in *Scenedesmus* sp. BHU1 from control to 0.4 M NaCl-treated cells reflects an important aspect of the adaptive response of microalgae to high salinity. This decrease is primarily attributed to ion competition, where high concentrations of Na^+^ in the surrounding environment compete with K^+^ for uptake by ion transporters in *Scenedesmus* sp. BHU1 cells. As Na^+^ becomes more abundant in saline culture, its increased presence inhibits the uptake of K^+^, leading to reduced K^+^ levels within the cells.

Although K^+^ is crucial for osmoregulation and maintaining cellular turgor pressure, the decrease in its levels suggests that *Scenedesmus* sp. BHU1 prioritizes the accumulation of other ions, such as Na^+^ and Ca^2+^, for osmotic regulation under increasing salt conditions (Figure 8E). Based on the findings, it was concluded that *Scenedesmus* sp. BHU1 regulates cellular homeostasis by modulating various symport and antiport channels. The observed changes in cation levels and their ratios suggest that *Scenedesmus* sp. BHU1 cells adjust their ion composition to optimize cellular functions and mitigate the detrimental effects of increasing salt stress.

### 3.8 Effect of NaCl on enzymatic antioxidant molecules of *Scenedesmus* sp. BHU1

**Figure 9:**
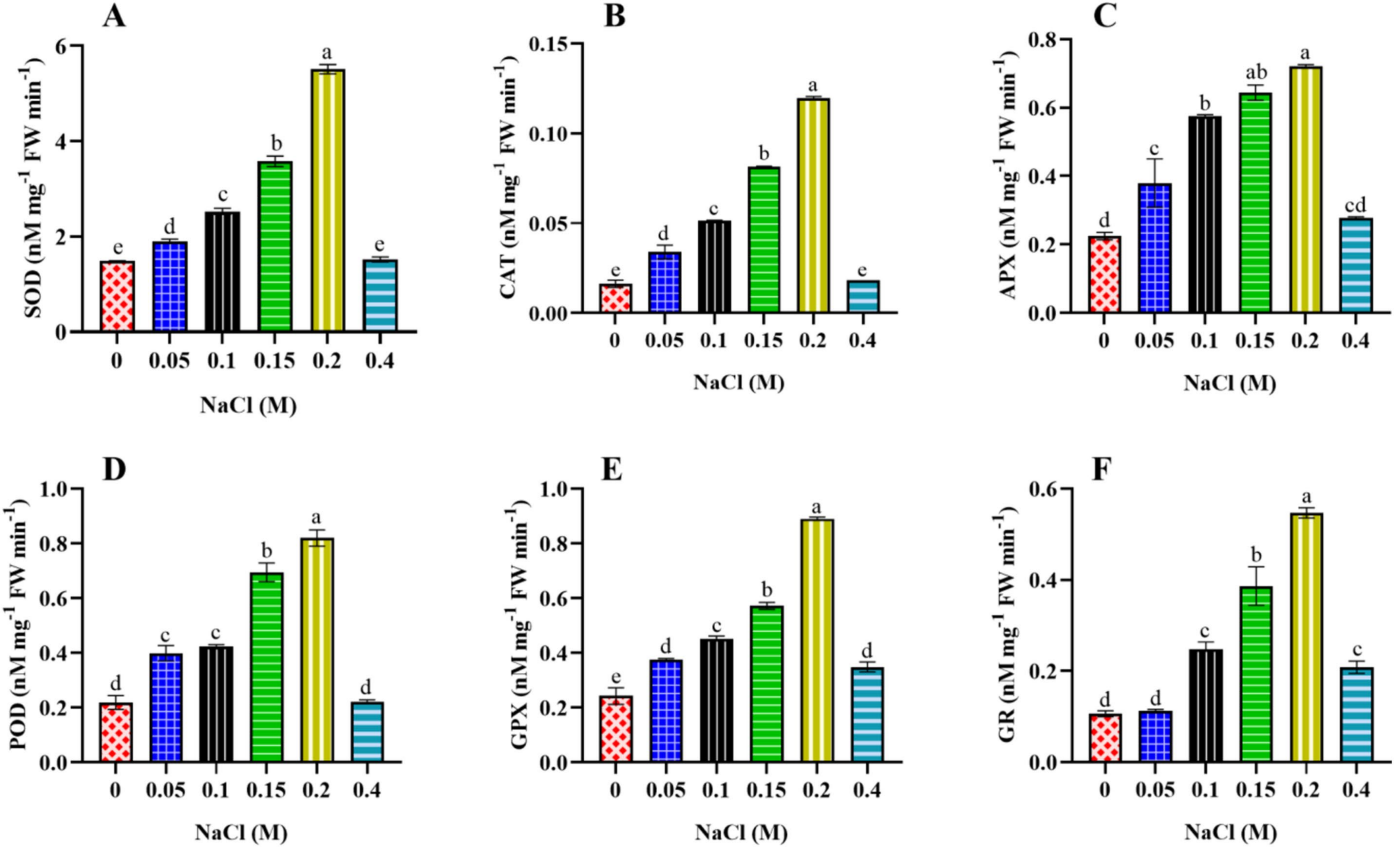
Effect of different concentrations of NaCl on the enzymatic antioxidant molecules of *Scenedesmus* sp. BHU1. The different panels represent: **(A)** superoxide dismutase (SOD), **(B)** catalase (CAT), **(C)** ascorbate peroxidase (APX), **(D)** peroxidase (POD), guaiacol peroxidase (GPX), and glutathione reductase (GR). Bars are the mean ± standard error of the mean (SEM) of three biological replicates (*n* = 3). Different lowercase alphabet letters (a–f) above the bars indicate the level of statistically significant differences, and the bars followed by the same alphabet letters refer to the statistically non-significant differences among treatment means at the *p* < 0.05 probability threshold (Tukey’s *post hoc* test).

The effect of salt on enzymatic antioxidant molecules, including SOD, CAT, APX, POD, GPX, and GR, can vary depending on the concentration of salt exposure and the specific characteristics of the organisms. SOD is an enzyme that plays a crucial role in scavenging superoxide radicals, which are generated as by-products of various metabolic processes. In our study, we found significantly increased (*p* < 0.05) levels of SOD from control to 0.2 M NaCl supplementation, with levels 3.7-fold higher in 0.2 M NaCl-treated cells as compared to control cells (Figure 9A). Similarly, an increase in SOD concentrations under increased NaCl supplementation was also reported in the cyanobacterium *Synechococcus* sp. PCC7942 (Verma et al., 2019). This is because increased salt concentrations can induce oxidative stress in cells by generating superoxide radicals, thereby increasing the need for SOD activity. However, excessive salt levels may also inhibit SOD activity due to changes in protein structure or interference with metal cofactors required for SOD function. That is why the SOD levels were decreased in *Scenedesmus* sp. BHU1 cells under 0.4 M NaCl content. Furthermore, CAT is another important antioxidant enzyme that catalyzes the decomposition of H_2_O_2_ into H_2_O and oxygen, thereby protecting cells from oxidative damage. In the current investigation, *Scenedesmus* sp. BHU1 showed a significant increase (*p* < 0.05) in CAT levels from control to 0.2 M NaCl-treated cells (Figure 9B). Furthermore, we noted that CAT levels decreased by 6.6-fold in 0.4 M NaCl-treated cells as compared to 0.2 M NaCl-treated cells. This indicates that moderate salt stress may increase CAT levels and activity as a protective response against oxidative stress induced by salt. However, prolonged exposure to high salt contents can lead to inhibition of CAT level and activity, possibly due to cellular damage or alterations in enzyme kinetics. Moreover, APX is an enzyme involved in the ascorbate-glutathione cycle, which helps detoxify H_2_O_2_ by using ascorbate as a specific electron donor. In our study, the APX content of *Scenedesmus* sp. BHU1 was 3.2-fold higher in 0.2 M NaCl-treated cells as compared to control cells (Figure 9C). Conversely, in 0.4 M NaCl-treated cells, the APX levels significantly decreased (*p* < 0.05) by 3.1-fold as compared to 0.2 M NaCl-treated cells. As a result, salt stress can modulate APX activity, with an increase in APX activity from control to 0.2 M NaCl concentrations, indicating moderate salt stress, possibly to mitigate oxidative damage. However, excessive salt levels may lead to decreased APX activity, potentially due to disruption of the ascorbate-glutathione cycle or inhibition of enzyme function. Additionally, POD enzymes encompass a diverse group of heme-containing enzymes involved in scavenging H_2_O_2_ and other ROS. Salt stress can influence POD activity depending on the microalgae species and the severity of the stress. In our study, the maximum level of POD was found in the control-grown cells, which was 3.7-fold higher than in the 0.2 M NaCl-treated cells (Figure 9D). Increasing salt stress may induce an increase in POD activity as part of the microalgae defense mechanism against oxidative stress. However, prolonged exposure to high salt concentrations can lead to decreased POD activity, possibly due to cellular damage or inhibition of enzyme function. Similar observations for all these enzymes were also reported in *A. fertilissima* under increasing concentrations of salt (Swapnil and Rai, 2018). Further, the GPX enzyme is a crucial component of the antioxidant defense system in cells across various organisms, including plants, animals, and microorganisms. Its primary function is to catalyze the reduction of H_2_O_2_ and organic hydroperoxides, thus protecting cells from oxidative damage caused by ROS. We have found a maximum GPX level in 0.2 M NaCl-treated cells, which was 3.6-fold higher than in control-grown cells (Figure 9E). This indicates that GPX enzymes catalyze the reduction of peroxides using glutathione (GSH) as a reducing agent. By scavenging ROS, GPX helps protect *Scenedesmus* sp. BHU1 cells from oxidative damage and maintain cellular integrity under salt stress conditions. The increased levels of GPX enzyme suggest an adaptive mechanism aimed at mitigating oxidative stress by efficiently detoxifying peroxides. Moreover, GR catalyzes the reduction of oxidized glutathione (GSSG) to its reduced form (GSH) using NADPH as a cofactor. This reaction is essential for maintaining a high ratio of GSH/GSSG, which is crucial for cellular antioxidant defense and detoxification processes. We observed a maximum level of GR in 0.2 M NaCl-treated cells, which was 5.1-fold higher than in control-grown cells (Figure 9F). This is because GR plays a vital role in maintaining redox homeostasis by catalyzing the reduction of GSSG to its GSH, which is essential for the proper functioning of antioxidant enzymes and defense mechanisms. Another reason may be that GR interacts with other components of the antioxidant defense system, such as GPX and the ascorbate-glutathione pathway, to efficiently scavenge ROS and maintain cellular redox balance. By regenerating GSH, GR enables GPX to detoxify peroxides and protect cells from oxidative stress-induced damage. The increased levels of GR from control to 0.2 M NaCl-treated cells suggest an adaptive strategy employed by microalgae to counteract salt-induced oxidative stress and preserve cellular integrity. Overall, the effect of salt on enzymatic antioxidant molecules includes both stimulatory and inhibitory effects, depending on the range of salt concentration and duration of exposure.

### 3.9 Effect of NaCl on lipidome of *Scenedesmus* sp. BHU1

**Figure 10:**
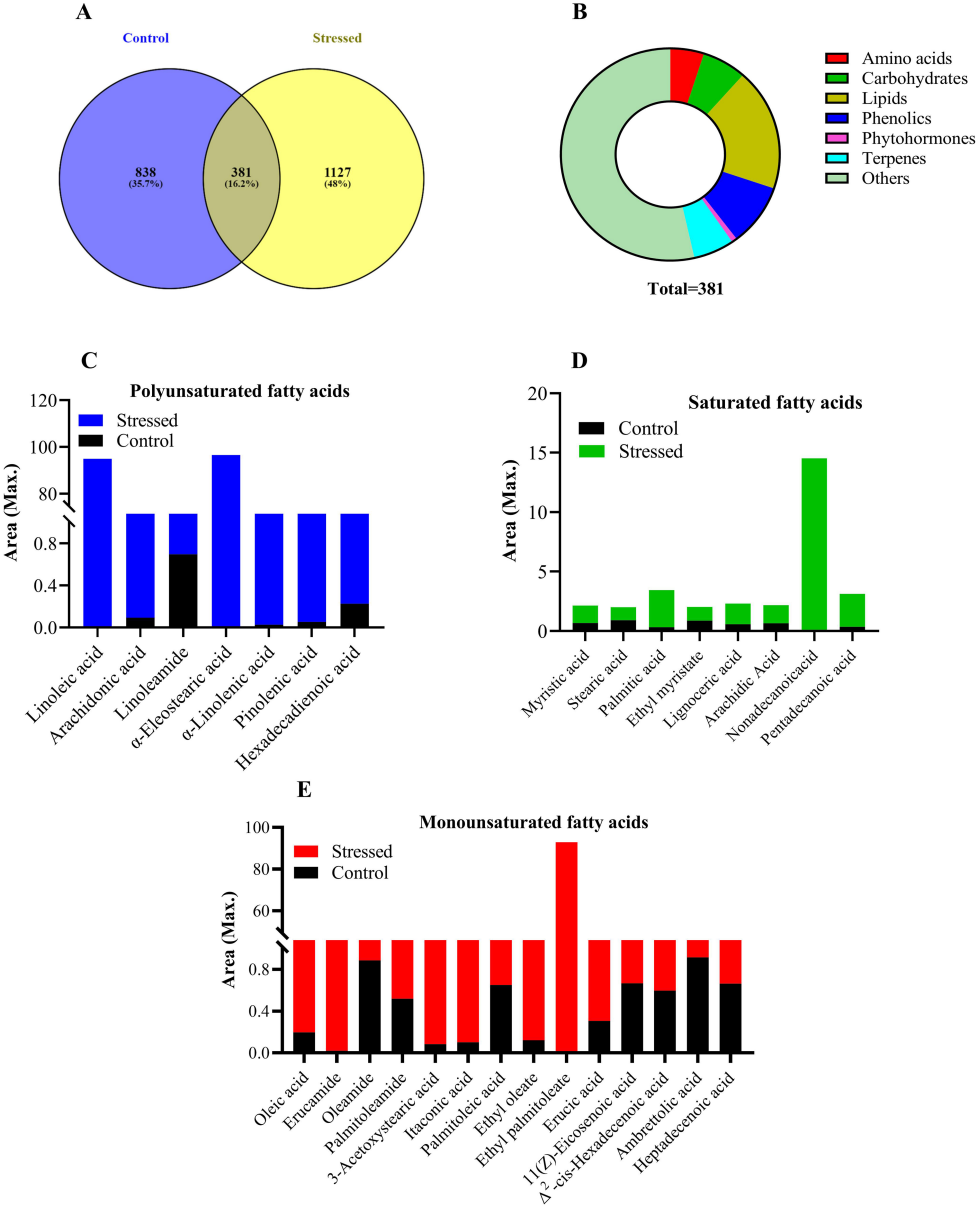
Metabolomics profiling of *Scenedesmus* sp. BHU1 under control and stressed (0.4 M NaCl) grown cultures. The different panels represent: **(A)** the number of total and common metabolites in control and stressed cells, **(B)** the major category of common metabolites in both control and stressed cells, **(C)** polyunsaturated fatty acids, **(D)** saturated fatty acids, and **(E)** monounsaturated fatty acids.

This section examined lipidomic alterations in *Scenedesmus* sp. BHU1 and their protective role in salt stress. The Venn diagram showed that we found 1,219 metabolites in control-grown cells and 1,508 metabolites in 0.4 M NaCl-treated (stressed) cells (Figure 10A). Among these metabolites, 381 (16.2%) are common in both the control and 0.4 M NaCl-treated cells. Further, we categorized these common metabolites into different groups, such as amino acids, carbohydrates, lipids, phenolics, phytohormones, and terpenes, and found that the majority were lipid-containing metabolites (Figure 10B). Figures 10C,D show that when *Scenedesmus* sp. BHU1 cells were stressed. They had 66 times more unsaturated fatty acids (UFAs) than the control group. Moreover, the content of saturated fatty acids (SFAs) increased by 6.1-fold in stressed cells as compared to control cells. The rise in UFAs can be seen as the microalgae’s way of protecting the membrane from salt-induced changes. Salt stress typically leads to membrane degradation, causing changes in membrane permeability, integrity, fluidity, and ion transport selectivity (Dao et al., 2024). The vital role of UFAs in adapting and tolerating salt stress is documented, primarily by shielding the plasma membrane and photosynthetic machinery. PUFAs and SFAs serve as building blocks for membranes, especially those of organelles. Earlier research has shown that reduced partitioning of PUFAs and long-chain SFAs hinders the cells’ capacity to generate new organelles, thereby restricting cell proliferation (Fal et al., 2022). In this study, the levels of both PUFAs and SFAs rose when exposed to salt stress. *Scenedesmus* sp. BHU1 increases the proportion of PUFAs in their membranes to maintain optimal fluidity under elevated salt contents. PUFAs are known for their ability to enhance membrane flexibility, allowing cells to adapt to salt stress. As a defense mechanism, *Scenedesmus* sp. BHU1 may synthesize more PUFAs to counteract the effects of oxidative stress. At the same time, they may also increase SFAs, which are more stable and less prone to oxidation, as a protective measure.

Further, it is noteworthy that under stressful conditions, the number of MUFAs (oleic acid; C18:1) increased by 25-fold as compared to the control, whereas PUFAs (linoleic acid; C18:2) experienced a 94-fold increase in cells subjected to stress. However, there was a 39-fold increase in another PUFA (α-linolenic acid; C18:3) in the stressed cells. Earlier studies have shown that MUFAs (C18:1) increased by 30.74% in *Monoraphidium* sp. when supplemented with 0.17 M NaCl as compared to control cells (Qiao et al., 2021). An earlier study also suggests that the levels of PUFAs in *Synechococcus elongatus* increased by 60.05% when exposed to 0.2 M NaCl as compared to control cells (Cui et al., 2020). These results indicate that salt stress may lead to an elevation in enzymes involved in the synthesis of PUFAs (Yang et al., 2024). In our research, we observed a higher quantity of C18:2 and C18:3 acids than C18:1 acids in stressed *Scenedesmus* sp. BHU1 cells. This increase in PUFAs, compared to MUFAs, may represent a defensive response aimed at reducing oxidative damage. PUFAs are characterized by increased fluidity and flexibility, which aid in preserving membrane integrity and functionality during stressful conditions. The shift toward greater synthesis of C18:2 could also be related to changes in the activity of specific enzymes involved in fatty acid desaturation (Δ^12^ desaturase; oleate desaturase). For instance, the Δ^12^ desaturase enzyme, which converts C18:1 to C18:2 by adding a double bond to oleic acid at position 12, might be upregulated under salt stress, suggesting an adaptive metabolic shift.

Furthermore, our study revealed elevated levels of C18:1 and C18:3 as compared to palmitic acid (C16:0) and stearic acid (C18:0) in *Scenedesmus* sp. BHU1 cells under salt stress. As a result, oleic acid, a MUFA, is synthesized from stearic acid (C18:0) via the action of the stearoyl-CoA desaturase enzyme. Its increased levels suggest an upregulation of this desaturase, which is a common response to salt stress. The presence of a double bond in C18:1 enhances membrane fluidity, which is crucial for maintaining cellular functions under elevated salt stress. This adaptation helps cells better cope with the osmotic stress induced by high salinity. Oleic acid acts as the primary product of fatty acid synthesis, which generates omega-3 fatty acids such as C18:3, eicosapentaenoic acid (C20:5), and others. This category of fatty acids plays a significant role in both biotic and abiotic stress responses. The PUFA, such as C18:3, is derived from C18:2 through further desaturation, a process involving the enzyme Δ^15^-desaturase. Linolenic acid plays a significant role in maintaining membrane fluidity and function, particularly under abiotic stress conditions. They are also involved in producing signaling molecules that mediate stress responses (Liang et al., 2023). SFAs such as C16:0 are common components of cell membranes. Their reduced levels in salt-stressed cells indicate a shift in fatty acid synthesis toward more unsaturated forms. SFAs tend to make cell membranes more rigid. Under stress conditions, cells benefit from increased membrane fluidity, which can be achieved by reducing SFA such as C16:0. Another SFA, such as C18:0, serves as a precursor for the production of C18:1 fatty acid. Its lower levels in salt-stressed cells suggest a rapid conversion to UFAs, which are more beneficial for stress adaptation. The enzymatic conversion of C18:0 to C18:1 is likely upregulated, reflecting the cells’ metabolic adjustment to enhance the synthesis of fatty acids that support stress resilience.

Additionally, the accumulation of fatty acids in microalgae under stress varies with the growth stage. Teh et al. (2021) found that salt stress increased the levels of C18:1 and C18:2 in *Chlorella vulgaris* during the mid and late stationary phases. These fatty acids can be easily converted into C18:3n3 by *ω*-3/ω-6 FAD enzymes, which are crucial for modulating growth and stress responses. Consequently, C18:3 serves as a precursor in the oxylipin pathway, which involves the synthesis of jasmonic acid (JA). The C18:3-derived hydroperoxy fatty acids (HPFAs) undergo further enzymatic conversions, including the action of allene oxide synthase (AOS) and allene oxide cyclase (AOC), ultimately producing JA and its derivatives. JA, as a plant growth regulator, influences algal growth and development, including protein synthesis, fatty acid metabolism, and stress resistance. This regulatory role is highlighted by the fact that applying methyl jasmonate (JA-Me) at a concentration of 100 μM enhanced the tolerance of *Scenedesmus incrassatulus* to the toxic effects of salinity under 0.175 M NaCl conditions (Fedina and Benderliev, 2000). Moreover, C18:3 has been linked to the activation of NADPH oxidase, a significant ROS generator in plants and microalgae. This study further supports this connection, as the increased ROS accumulation observed may be attributed to the activation of NADPH oxidase by the accumulated C18:3.

Moreover, other SFAs, such as pentadecanoic acid or pentadecylic acid (C15:0), ethyl myristate or ethyl tetradecanoate (C16:0), nonadecanoic acid or nonadecylic acid (C19:0), arachidic acid or icosanoic acid (C20:0), and lignoceric acid or tetracosanoic acid (C23:0), were also increased in stressed-grown cells. The increases were 9.1-, 1.3-, 240-, 2.5-, and 3.4-fold higher, respectively, as compared to the control-grown cells. This significant rise in SFAs indicates that, under salt stress, microalgae undergo lipid remodeling to maintain cell integrity and function. The increased levels of these specific fatty acids suggest a shift toward producing more saturated and longer-chain fatty acids (LCFAs). LCFAs are often associated with increased membrane stability and energy storage. The presence of LCFAs such as C19:0, C20:0, and C23:0 implies that fatty acid elongation pathways are upregulated (Fal et al., 2022). This enzymatic process involves the addition of two-carbon units to shorter fatty acid chains, which may be enhanced under stress conditions. The accumulation of LCFAs could be a strategy for energy storage. These fatty acids can be stored as triacylglycerols (TAGs) and mobilized when the stress condition is alleviated, providing a reservoir of energy for recovery and growth. Furthermore, these SFAs contribute to the rigidity and stability of cellular membranes, which is particularly important under salt stress. Maintaining membrane integrity is crucial for cellular survival, and the increased levels of these fatty acids likely help stabilize cell membranes against osmotic pressure and ion toxicity. Microalgae respond to salt stress by increasing LCFAs, which help stiffen membranes and reduce salt permeability (Kondo et al., 2016). These LCFAs are crucial precursors in cutin and wax elongation processes, highlighting their significance in adapting to environmental stressors. The fatty acid composition of microalgae is shaped by metabolic pathways activated during salinity stress, indicating a regulatory mechanism for tolerance. Our study on *Scenedesmus* sp. BHU1 reveals its coping strategy of elevating SFAs such as C19:0, C20:0, and C23:0 in salt-stressed cells.

Besides SFAs, we observed a higher number of MUFAs than PUFAs; however, the amount of PUFAs was 1.4-fold higher than that of MUFAs in salt-stressed cells of *Scenedesmus* sp. BHU1 (Figures 10C,E). Exposure of *Scenedesmus* sp. BHU1 cells to 0.4 M NaCl resulted in alterations in the distribution of MUFAs and PUFAs. The dominant MUFAs identified were itaconic acid (C5:1), oleic acid (C18:1), ethyl palmitoleate (C18:1ω9), 3-acetoxystearic acid (C20:1), erucamide (C22:1), and erucic acid (C22:1ω9) in salt-treated cells. C5:1 is typically produced by fungi but can also be synthesized by other microorganisms. Its increased levels in salt-stressed microalgae could indicate alterations in the citric acid cycle or other metabolic pathways due to stress (Wang et al., 2021). C18:1 and C18:1ω9 have known roles in membrane fluidity and signaling processes, suggesting adaptations to maintain membrane integrity and function under salt stress. C20:1 is a modified form of stearic acid, with elevated levels potentially related to lipid modification processes under stress conditions, affecting membrane properties. The presence of C22:1 and C22:1ω9, both LCFAs, suggests changes in lipid metabolism, possibly linked to the synthesis of lipid-based protective compounds or alterations in membrane properties. Additionally, the presence of these LCFAs, along with erucamide, indicates potential alterations in lipid biosynthesis pathways and membrane lipid composition under salt stress conditions. The dominant PUFAs identified in salt-treated *Scenedesmus* sp. BHU1 cells were linoleic acid (C18:2:ω9,12), pinolenic acid (C18:3:Δ5,9,12), α-eleostearic acid [(C18:3:9Z,11E,13E)-octadeca-9,11,13-trienoic acid], α-linolenic acid (C18:3:ω9,12,15), and arachidonic acid (C20:4:Δ5,8,11,14). The increased levels of linoleic acid under salt stress suggest that microalgae may be enhancing their membrane fluidity and flexibility, which is crucial for maintaining cellular integrity in elevated salinity (Fal et al., 2022). Similarly, elevated levels of pinolenic acid may indicate an adaptive response to mitigate oxidative stress and inflammation caused by high salt concentrations, thereby protecting cellular components. Moreover, the increase in α-eleostearic acid suggests alterations in lipid metabolism aimed at enhancing the stability and protective functions of cell membranes under salt stress conditions. Additionally, higher levels of α-linolenic acid may help maintain or restore the proper functioning of cellular membranes by countering the rigidity imposed by high salt concentrations, thus ensuring optimal cellular processes (Baccouch et al., 2023). Furthermore, the increased production of arachidonic acid under elevated salt stress likely reflects the activation of stress response pathways, including the production of signaling molecules that help manage the stress and initiate adaptive responses.

In this study, the fatty acid profile of *Scenedesmus* sp. BHU1 exhibited a marked increase in SFAs, particularly myristic acid, palmitic acid, pentadecanoic acid, and nonadecanoic acid, along with trace levels of stearic acid, ethyl myristate, and lignoceric acid in stressed cells as compared to control-grown cells. Moreover, among MUFAs, the highest concentration was observed for ethyl palmitoleate, followed by erucamide, 3-acetoxystearic acid, itaconic acid, and oleic acid. Similarly, among PUFAs, the highest concentration was detected for α-eleostearic acid, followed by linoleic acid, α-linolenic acid, and pinolenic acid in stressed cells as compared to the control cells. Similarly, Saadaoui et al. (2024) reported elevated levels of MUFAs and PUFAs in *Tetraselmis subcordiformis* when exposed to salinity stress. This shift in the fatty acid composition under increased salt conditions suggests that salinity may significantly influence the cell membrane composition of *Scenedesmus* sp. BHU1. Interestingly, this study revealed that the increased levels of SFAs, MUFAs, and PUFAs in stressed cells each have distinct biotechnological applications across various industries, owing to their unique chemical properties. SFAs are commonly used in the production of biodiesel, where they provide good oxidative stability, a crucial factor for the fuel’s shelf life. However, their higher melting points can lead to poor cold flow, a property that is particularly important to consider in colder climates. Beyond the energy sector, SFAs find extensive use in the food industry, particularly in the production of margarine and other solid fats, where they contribute to the texture and stability of these products.

Additionally, SFAs, such as stearic acid, are integral to the formulation of soaps, lotions, and creams, acting as surfactants and emulsifying agents that help stabilize and thicken these products. Similarly, MUFAs, particularly oleic acid, are extensively used in functional foods that promote heart health and are incorporated into dietary supplements due to their potential to lower bad cholesterol levels and reduce inflammation (Shen et al., 2023). In the field of biofuels, MUFAs are ideal candidates for biodiesel production, offering a good balance between oxidative stability and low-temperature fluidity. As a result, biodiesel produced from MUFAs tends to exhibit better cold flow properties than those derived from SFAs. Beyond their role in energy and nutrition, MUFAs are widely used in skin care products for their moisturizing properties, which help improve skin barrier function and hydration, making them popular in creams, lotions, and oils.

Similarly, PUFAs, including omega-3 and omega-6 fatty acids, are essential nutrients with a broad range of health benefits. These fatty acids are incorporated into supplements and functional foods to support cardiovascular, brain, and immune health. In the pharmaceutical industry, PUFAs play a crucial role in developing treatments for managing chronic diseases such as cardiovascular diseases, arthritis, and mental health disorders. Moreover, PUFAs are vital for the health and growth of fish and livestock. They are added to animal feed to improve the nutritional quality of farmed fish and meat, ultimately benefiting human health. In the skincare industry, PUFAs are valued in anti-aging and skin regeneration products because they maintain skin elasticity and moisture. They help protect the skin from damage and support its natural repair processes.

### 3.10 Effect of NaCl on transcriptomics of *Scenedesmus* sp. BHU1

**Figure 11:**
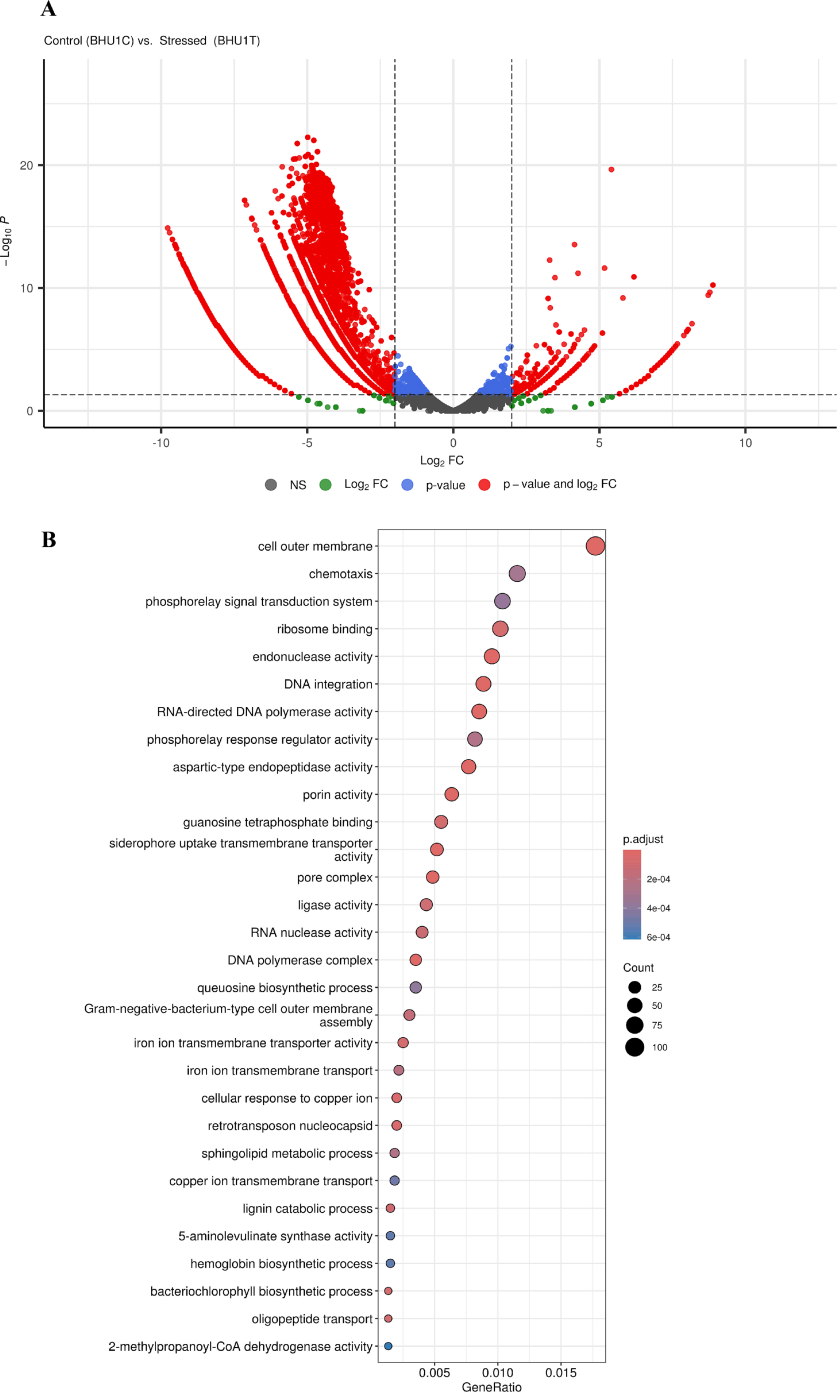
Transcriptome sequencing analysis of *Scenedesmus* sp. BHU1. The different panels represent: **(A)** a volcano plot displaying the differentially expressed genes and **(B)** a dot plot displaying the gene ontology for differentially expressed genes of *Scenedesmus* sp. BHU1 control vs. stressed cells. BHU-1C and BHU-1 T represent control and stressed cells (0.4 M NaCl), respectively.

To understand how salinity affects the synthesis and metabolism of bioactive substances in *Scenedesmus* sp. BHU1, a comparative transcriptomic analysis was conducted between control and salt-stressed cells (0.4 M NaCl). After removing linker sequences and low-quality reads, a total of 25,103,729 sequences were obtained for the control cells and 13,734,187 sequences for the stressed cells. The analysis revealed that 2,281 proteins were covered by more than 90% of their protein lengths, and 3,089 proteins were represented by nearly full-length transcripts with over 80% alignment coverage. Additionally, 808 proteins matched a Trinity transcript with alignment coverage between 80 and 90% of their protein lengths, underscoring the comprehensive nature of the transcriptome assembly. Figure 11A illustrates the distribution and levels of differentially expressed genes (DEGs), highlighting changes in gene expression. A dot plot of gene ontology analysis identified carbon, membrane, and lipid metabolism as the most represented pathways (Figure 11B). In this figure, the significance of enrichment is indicated by the darkness of the bubble color, with redder bubbles representing higher significance. The gene ratio, reflected by the size of the bubbles, indicates the degree of enrichment—the larger the ratio, the greater the enrichment. Larger bubbles correspond to a higher number of genes enriched in the specific term. Among these differentially expressed genes, a network plot in Supplementary Figure S2 displays the gene ontology for those involved in photosynthesis, CO_2_ fixation, chlorophyll synthesis and degradation, carbohydrate accumulation, the tricarboxylic acid (TCA) cycle, and glycolysis, illustrating the interconnectedness of these metabolic processes.

**Figure 12:**
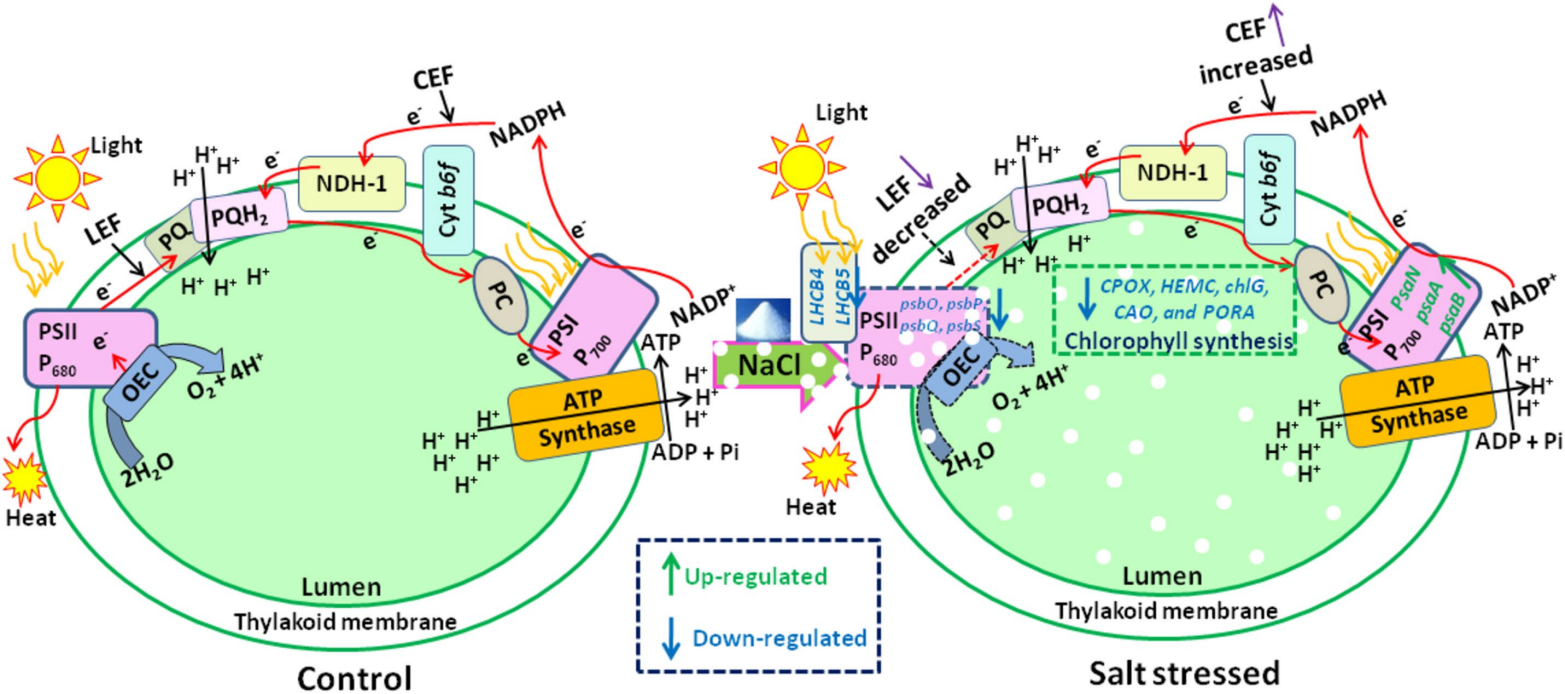
A schematic diagram summarizing the changes in the photosynthetic system of *Scenedesmus* sp. BHU1 under salt stress. The various abbreviations represent: PSII (photosystem II), H^+^ (proton), e^−^ (electron), OEC (oxygen-evolving complex), LEF (linear electron flow), PQ (plastoquinone), PQH_2_ (reduced plastoquinone), Cyt b_6_f (cytochrome b_6_f complex), PC (plastocyanin), PSI (photosystem I), ADP (adenosine diphosphate), Pi (inorganic phosphate), ATP (adenosine triphosphate), NADP^+^ (nicotinamide adenine dinucleotide phosphate), NADPH (nicotinamide adenine dinucleotide phosphate hydrogen), and CEF (cyclic electron flow). In this diagram, the dotted line represents the damaged system, whereas the solid line represents the functional system.

To further investigate the mechanisms of photosystem variation under salt stress, genes involved in the metabolic pathways of photosynthesis and chlorophyll synthesis were studied (Figure 12). Further analysis of the DEGs in the aforementioned processes revealed that the relative gene expressions of photosystem II oxygen-evolving enhancer protein 1 (*PsbO*), oxygen-evolving enhancer protein 3 (*PsbQ*), and PsbP domain-containing protein 7 (*PsbP5*) were downregulated in salt-stressed cells (Supplementary Table S3). Additionally, high salinity downregulated the gene expression of PSII components, including *LHCA4*, *atpA*, *atpH*, *atpG*, *LHCQ*, *LHCA*, *Lhcb2-1*, *LHCB4*, *LHCA*, *LHCB5*, *LHCA5*, *LHCA3*, *petF3*, and *psbY*. Moreover, genes encoding other PSII proteins, such as *psbO*, *psbP5*, *psbP3*, *psbQ*, *psbS*, and *psbY,* were downregulated under elevated salt stress. These proteins are peripheral components of the OEC, crucial for sustaining the activity of PSII (Li et al., 2022). LHCs capture and direct excitation energy into the photoreaction system, which powers photosynthesis (Neilson and Durnford, 2010). The downregulation of the LHC gene suggests a reduced capacity for harvesting and transferring excitation energy. Additionally, the downregulation of F-type ATPase subunits [γ (*atpA* and *atpG*) and δ (*atpH*)] of the LEF indicates a reduction in ATP synthesis under high salt conditions. Furthermore, the downregulation of *PGK*, *TKTA*, *fbp*, *gapA*, *SBPase*, and *rbcL* in the C_3_ photosynthetic cycle suggests a significant reduction in the ability of *Scenedesmus* sp. BHU1 to sustain the CO_2_ fixation rate in stressed cells. Elevated salinity in microalgae causes ionic and osmotic stress, disrupting various metabolic pathways such as photosynthesis and CO_2_ fixation (Perrineau et al., 2014). Additionally, the genes *CPOX*, *HEMC*, *chlG*, *CAO*, and *PORA* are crucial in the chlorophyll synthesis pathway (Li et al., 2022). In this study, these genes were downregulated in stressed cells, consistent with previous findings that showed a decrease in photosynthetic efficiency (Fv/Fm, YII, Fv, and ETR_max_) under salt stress conditions. In contrast, PSI-related genes, including *psaA*, *psaB*, *LHCA5*, and *PsaN*, were upregulated under increased salt content.

Further, according to the DEG analysis, metabolic pathways related to carbohydrate accumulation, such as glycolysis, the TCA cycle, and polysaccharide synthesis (including starch and cellulose), were upregulated under salt stress (Supplementary Table S3). Glycolysis is believed to play a beneficial role in the development and adaptation of microalgae to environmental stresses (Song et al., 2020). Compared to control cells, the genes encoding hexokinase-1 (*HK*) and pyruvate kinase (*PK*) were significantly upregulated in stressed *Scenedesmus* sp. BHU1 cells were downregulated, whereas the genes encoding fructose bisphosphate aldolase (*ALDO*) and phosphoglycerate kinase (*PGK*) were in the glycolytic pathway. This finding suggests that the glycolysis process beyond glucose 6-phosphate may be impeded, potentially leading to a temporary accumulation of intermediate products such as fructose 6-phosphate and glucose 6-phosphate. These could then be converted into glucose 1-phosphate to synthesize related polysaccharides. The TCA cycle is crucial for cellular energy metabolism and the supply of carbon skeletons (Liu et al., 2018). A distinct trend was observed where key enzymes associated with the TCA cycle were upregulated under high salinity stress (Supplementary Table S3). This includes genes encoding rate-limiting enzymes such as citrate synthase 1 (*citA*), isocitrate dehydrogenase kinase/phosphatase (*aceK*), and the 2-oxoglutarate dehydrogenase E1 component (*sucA*), which supply more ATP and NADPH for other metabolic pathways. Polysaccharides such as cellulose, sucrose, and trehalose are crucial for counteracting biotic stresses in microalgae (de Jaeger et al., 2018). In salt-treated *Scenedesmus* sp. BHU1, glucose-1-phosphate adenylyltransferase (*glgC*) and starch synthase 3 (*GLGA3*), which are involved in converting ADP-glucose into starch (Schreier et al., 2021), were upregulated. Additionally, sucrose synthase 2 (*SUS4*), essential for cell wall synthesis (Jeong et al., 2017), was upregulated. The expression patterns of DEGs aligned with the observed increase in starch, cellulose, and glucose content under salt stress.

Additionally, DEGs involved in fatty acid metabolism were identified in *Scenedesmus* sp. BHU1 is generally recognized as similar to the fatty acid synthesis pathways observed in other microalgae. Notably, under salt stress conditions, genes encoding key enzymes such as acetoacetyl-CoA synthetase (*acsA*), 3-oxoacyl-[acyl-carrier-protein] synthase 1 (*fabB*), 3-oxoacyl-[acyl-carrier-protein] reductase (*fabG*), 3-oxoacyl-[acyl-carrier-protein] synthase 2 (*fabF*), beta-ketoacyl-[acyl-carrier-protein] synthase III (*fabH*), and 3-hydroxyacyl-[acyl-carrier-protein] dehydratase (*babZ*) were upregulated as compared to control cells (Supplementary Table S3). This upregulation is closely associated with the elevated expression levels of 3-ketoacyl-CoA synthase 7 (*KCS*) and long-chain acyl-CoA synthetase 4 (*ACSL*), which are crucial for the elongation cycle of LCFAs in the endoplasmic reticulum (He et al., 2019). The increased expression of *KCS* and *ACSL* further indicates that high salinity conditions enhance the synthesis of LCFAs. Moreover, the upregulation of stearoyl-CoA desaturase (*DesC*), a key enzyme responsible for the synthesis and metabolism of UFAs by catalyzing the conversion of 16:0 and 18:0 into 16:1 and 18:1n9c (Song et al., 2020), is consistent with the increased content of MUFAs, specifically C16:1 and C18:1n9c, observed in salt-stressed cells. This interconnected series of gene expressions and enzyme activities suggests that salt stress promotes the production of LCFAs and enhances the synthesis of UFAs, thereby increasing membrane fluidity and helping protect the cell from potential damage (Li et al., 2021).

### 3.11 Correlation analysis between photosynthetic parameters and heat map of lipidome

Correlation analysis was conducted to assess the direction and degree of the relationships between various physiological characteristics of *Scenedesmus* sp. BHU1 under salt stress (Supplementary Figure S3A). This approach helps us understand how changes in salt concentration affect the various physiological properties of *Scenedesmus* sp. BHU1. For slow kinetics and RLC parameters, Fv/Fm exhibited a positive correlation with Y(II), Y(NPQ), NPQ, qN, qP, qL, qE, qI, qNP, α, I_k_, and ETR_max_, while being negatively correlated with Y(NO). This suggests that a coordinated response exists in the physiological behavior of *Scenedesmus* sp. BHU1 to variations in these parameters under salt stress. When these parameters exhibit positive correlations, it indicates that as the values of Fv/Fm increase or decrease, the values of the other parameters also tend to increase or decrease. In our results, increased salt content likely creates unfavorable conditions for the microalgae, leading to inefficient electron transport and photosynthesis. This ultimately results in decreased photochemical efficiency and reduced effective energy utilization. On the other hand, the negative correlation with Y(NO) indicates an inverse relationship between Fv/Fm and Y(NO). Specifically, decreased values of Fv/Fm are associated with increased Y(NO) values in stressed *Scenedesmus* sp. BHU1. This suggests that increased salt content leads to higher non-regulated energy dissipation, meaning more energy is lost as heat rather than being utilized for beneficial biochemical processes.

Furthermore, Fv/Fm exhibited positive correlations with fast kinetics parameters, including TR_O_/RC, ET_O_/RC, RC/CS_O_, ABS/CS_O_, TR_O_/CS_O_, ET_O_/CS_O_, φ_Po_, ѱo, φ_Eo_, φ_Ro_, PI_ABS_, and PI_CSo_. Conversely, it displayed negative associations with ABS/RC, DI_O_/RC, RE_O_/RC, DI_O_/CS_O_, δ_Ro_, and φ_Do_. These correlations suggest that a decrease in Fv/Fm value in stressed *Scenedesmus* sp. BHU1 cells are associated with lower photosynthetic efficiency, inefficient utilization of light energy, and possibly a less active photosynthetic apparatus. Furthermore, the inverse relationship implies that a decrease in Fv/Fm is linked to an increase in energy dissipation per reaction center and per cross-section. This suggests that *Scenedesmus* sp. BHU1 has likely adapted to survive salt stress by modifying its photosynthetic strategy. This adjustment may involve a trade-off between maximizing energy absorption (as indicated by higher values of ABS/RC and related parameters) and optimizing photosynthetic efficiency (as indicated by Fv/Fm). The negative correlations suggest that, during acclimatization, the organism may prioritize energy capture over efficient energy conversion, potentially due to salt stress. The observed correlations in response to salt stress indicate that *Scenedesmus* sp. BHU1 undergoes acclimatization processes.

Additionally, the increased levels of both SFAs and UFAs in salt-stressed *Scenedesmus* sp. BHU1 cells are shown by the heat map (Supplementary Figure S3B). The heat map effectively illustrates the differential expression of various fatty acids between control and salt-stressed cells. It visually represents how lipid profiles are altered in response to salt stress, highlighting the specific fatty acids that are upregulated under these conditions. SFAs are known to increase the rigidity and stability of cellular membranes. Under salt stress, maintaining membrane integrity is crucial to counteract osmotic pressure and ion toxicity. The increased levels of SFAs likely contribute to strengthening cell membranes, providing structural support, and reducing permeability to salt ions. SFAs can be stored as TAGs and utilized when the stress conditions are alleviated. This serves as a reservoir of energy for recovery and growth once favorable conditions return. UFAs play a crucial role in maintaining membrane fluidity and flexibility. In high-salinity environments, increased levels of UFAs help counteract the rigidity imposed by SFAs, ensuring optimal membrane functionality and flexibility. This balance is essential for maintaining cellular processes and nutrient transport across the membrane. These fatty acids contribute to overall stress tolerance by protecting cellular components and ensuring membrane stability. The simultaneous increase in SFAs and UFAs indicates a complex adaptive mechanism where microalgae adjust their lipid composition to maintain cellular integrity and function under salt stress. The balance between membrane rigidity (SFAs) and fluidity (UFAs) is crucial for optimal cell survival. The accumulation of both SFAs and UFAs suggests a strategic approach to energy management, with SFAs serving as energy reserves and UFAs ensuring membrane functionality and stress signaling.

## 4 Conclusion

Based on the results of this investigation, we conclude that elevated salinity stress altered the net photosynthetic productivity, primarily through a reduction in PSII photochemical yield. These alterations can be attributed to a shift in the electron donor and acceptor sides of PSII. Furthermore, the increased salt content is detrimental to the redox poise of the photosynthetic ETC, leading to limitations in electron flow within PSII. The drop in the OJIP curve of salt-treated cells indicates the oxidative effect of salt, which causes a decline in the electron donor and acceptor pools of PSII. Nevertheless, *Scenedesmus* sp. BHU1 cells attempt to maintain intracellular redox poise by regulating energy flux per reaction center and cross-section. Therefore, it can be concluded that elevated salt stress drastically decreases PSII quantum efficiency, adversely affecting its functional framework. In light of these findings, it becomes evident that PSII-dependent LEF decreases, while PSI-driven CEF is activated as part of the acclimatization process to protect *Scenedesmus* sp. BHU1 from salt-induced photoinhibition. The flow cytometry graphs depict the viability assay of *Scenedesmus* sp. BHU1, showing a gradual decrease in viability under increased salt stress. Our observations revealed increased levels of biochemical attributes, such as stress biomarkers, osmoprotectants, and enzymatic antioxidants, which aim to scavenge ROS.

Additionally, intracellular levels of Na^+^ and Ca^2+^ rose, while K^+^ levels decreased, indicating cellular homeostasis under increased salt content. UHPLC-HRMS-based lipidomics analysis confirmed that higher salt stress induces the overaccumulation of MUFAs and PUFAs over SFAs. In response to salt stress, the genes involved in PSI, glycolysis, the TCA cycle, starch metabolism, sucrose metabolism, lipid accumulation, and cellulose biosynthesis were upregulated to counteract the detrimental effects of salt.

While our study provides valuable insights into the adaptation mechanisms of freshwater microalgae to salt stress, it is important to acknowledge that focusing on a single species and strain may limit the generalizability of our findings. Freshwater microalgae are highly diverse, with significant variability in adaptation mechanisms across different species and strains. Therefore, the results obtained from *Scenedesmus* sp. BHU1 may not fully capture the broader adaptation mechanisms of microalgae observed in other freshwater species under similar conditions. This limitation highlights the need for further studies involving a diverse range of freshwater microalgal species and strains to better understand the diversity and adaptability of microalgae in response to salt stress.

## Data Availability

The raw data supporting the conclusions of this article will be made available by the authors, without undue reservation.

## Footnotes

^1^https://github.com/trinityrnaseq/trinityrnaseq/wiki
