# Supplementary material for: Unlocking the adaptation mechanisms of the oleaginous microalga Scenedesmus sp. BHU1 under elevated salt stress: a physiochemical, lipidomics and transcriptomics approach
Source: Front Microbiol. 2024 Nov 18;15:1475410. doi: 10.3389/fmicb.2024.1475410 (PMC11610451; doi:10.3389/fmicb.2024.1475410)
Supplement: Supplementary file 1 [file Data_Sheet_1.doc]

**Unlocking the adaptation mechanisms of the oleaginous microalga *Scenedesmus* sp. BHU1 under elevated salt stress: A physiochemical, lipidomics and transcriptomics approach**

Rahul Prasad Singh^1^, Priya Yadav^1^, Himani Sharma^1^, Ajay Kumar*^2^, Abeer Hashem^3^, Elsayed Fathi Abd_Allah^4^, and Rajan Kumar Gupta*^1^

^1^Laboratory of Algal Research, Department of Botany, Institute of Science, Banaras Hindu University, Varanasi-221005, India.

^2^Amity Institute of Biotechnology, Amity University, Noida 201303, India.

^3^Botany and Microbiology Department, College of Science, King Saud University, P.O. Box. 2460, Riyadh 11451, Saudi Arabia.

^4^Plant Production Department, College of Food and Agricultural Sciences, King Saud University, P.O. Box. 2460, Riyadh 11451, Saudi Arabia.

***Corresponding Authors:**

1. Rajan Kumar Gupta ([rajang.bot@bhu.ac.in](mailto:rajang.bot@bhu.ac.in))

2. Ajay Kumar ([ajaykumar_bhu@yahoo.com](mailto:ajaykumar_bhu@yahoo.com))

**Supplementary Tables and their captions**

**Supplementary Table S1. Definition and calculation formulas of photosynthetic parameters of PSII applied for slow kinetics and RLC.**

| **Definition of parameters** | **Formula of parameters** |
| --- | --- |
| Maximum photochemical **efficiency** | Fv/Fm = Fm−Fo/Fm |
| Effective photochemical **efficiency** | Y(II) = Fmʹ−F/Fmʹ |
| Variable fluorescence of dark-adapted cells | Fv = Fm−Fo |
| Non-regulated heat dissipation and fluorescence emission | Y(NO) = F/Fm |
| Light-induced non-photochemical fluorescence quenching | Y(NPQ) = F/Fmʹ−F/Fm |
| Stern-Volmer type non-photochemical fluorescence quenching | NPQ = Fm/Fmʹ−1 |
| Coefficient of photochemical fluorescence quenching based on the puddle model | qP = Fmʹ−F/Fmʹ−Foʹ |
| Coefficient of photochemical fluorescence quenching assuming interconnected PSII antennae based on the lake model | qL = qP∙Foʹ/F |
| Coefficient of non-photochemical fluorescence quenching | qN = 1−Fmʹ−Foʹ/Fm−Fo |
| Fast phase high-energy state fluorescence quenching | qE = qf = 1−a/c |
| Slow phase photoinhibitory fluorescence quenching | qI = qs = 1−d/Fv |
| Total non-photochemical fluorescence quenching | qNP = 1−a/Fv |
| Initial slope of the rapid light response curve | α (electrons/photons) = 1/c |
| Maximum rate of electron transfer | ETRmax (µmol electrons/m^-2^s^-1^) = 1/b+2∙√a∙c |
| Minimum saturating light intensity | Ik (µmol photons/m^-2^s^-1^) = c/b+2∙√a∙c |

**Supplementary Table S2. Using data extracted from OJIP fluorescence transient curves, we defined and calculated photosynthetic parameters deduced by the JIP-test.**

| **Definition of parameters** | **Formula of parameters** |
| --- | --- |
| **(a)** **Extracted fundamental/basic fluorescence parameters** | |
| Fluorescence intensity at time t after onset of actinic light | Ft |
| Minimum fluorescence at 20 µs (When all PSII RCs are opened) | F_O_ |
| Fluorescence intensity at 0.3 ms at the K-phase of the OKJIP curve | F_K_ |
| Fluorescence intensity at 2 ms at the J-phase of the OKJIP curve | F_J_ |
| Fluorescence intensity at 30 ms at the I-phase of the OKJIP curve | F_I_ |
| Maximum fluorescence intensity (When all PSII RCs are closed) | F_M_ = F_P_ |
| Time (ms) to reach the maximum fluorescence intensity (F_M_) | t_FM_ |
| **(b) Fluorescence parameters generated from the extracted data** | |
| Maximum variable fluorescence | F_V_ = F_M_−F_O_ |
| Relative variable fluorescence at time 2 ms (J-phase) | V_J_ = (F_J_−F_O_)/(F_M_−F_O_) |
| Relative variable fluorescence at time 30 ms (I-phase) | V_I_ = (F_I_−F_O_)/(F_M_−F_O_) |
| Initial slope of the fluorescence induction curve normalized on the maximal variable fluorescence; it express the rate of electron trapping | M_O_ = 4∙(F_K_−F_O_)/(F_M_−F_O_) |
| **(c) Specific energy fluxes per Q_A_− PSII reaction center (RC)** | |
| Absorption flux per active RC | ABS/RC = M_O_∙(1/V_J_)∙(1/φ_Po_) |
| Trapping flux per active RC | TR_O_/RC = M_O_∙(1/V_J_) |
| Electron transport flux per active RC | ET_O_/RC = M_O_∙(1/V_J_)∙ѱo |
| Electron flux reducing end electron acceptors at the PSI acceptor side per RC | REo/RC = M_O_∙(1/V_J_)∙(1−V_I_) |
| Dissipation of excess energy per active RC | DI_O_/RC = (ABS/RC)−(TR_O_/RC) |
| Density of RC (Q_A_ reducing PSII RC) | RC/CSo = φ_Po_∙(V_J_/M_O_)∙F_O_ |
| **(d) Phenomenological energy fluxes per excited sample cross-section (CS)** | |
| Absorption flux per excited CS | ABS/CS_O_ = F_O_ |
| Trapping flux per excited CS | TR_O_/CS_O_ = ABS/CS_O_∙φ_Po_ |
| Electron transport flux per excited CS | ET_O_/CS_O_ = ABS/CS_O_∙φ_Po_∙ѱo |
| Dissipation of excess energy per excited CS | DI_O_/CS_O_ = (ABS/CS_O_)−(TR_O_/CS_O_) |
| **(e) Quantum efficiencies or** **energy flux ratios** |  |
| Photosynthetic integrity of primary PSII photochemistry | φ_Po_ = F_V_/F_M_ |
| Efficiency/probability with which a PSII-trapped electron is transferred further than Q_A_− into the ETC | ѱo = (1−V_J_) |
| Quantum yield of electron transport from Q_A_− to plastoquinone | φ_Eo_ = ѱo∙φ_Po_ |
| Efficiency/probability with which an electron is transferred from Q_B_ to PSI acceptors | δ_Ro_ = (1−V_I_)/(1−V_J_) |
| Quantum yield of energy dissipation | φ_Do_ = F_O_/F_M_ |
| Quantum yield of reduction in end electron acceptors at the PSI acceptor side | φ_Ro_ = RE_O_/ABS = [1−(F_O_/F_M_)]∙ψo ∙δ_Ro_ |
| **(f) Density of RC** |  |
| Q_A_− reducing per RC, reflecting density of RC at t = t_FM_ | RC/ABS = φ_Po_·(V_J_/M_o_)·(ABS/RC) |
| Q_A_− reducing RC per CS, reflecting density of RC at t = t_FM_ | RC/CSo = φ_Po_·(V_J_/M_o_)·(ABS/CSo) |
| **(g) Performance index** |  |
| Performance index (potential) for energy conservation from photons absorbed by PSII to the reduction of intersystem electron acceptors | PI_ABS_=(RC/ABS)·[φ_Po_/(1−φ_Po_)]·[ψo/(1−ψo)] |
| Performance index based on cross section at t = t_FM_ | PI_CSo_ =(RC/CSo)·[φ_Po_/(1−φ_Po_)]·[ψo/(1−ψo)] |

**Supplementary Table S3.** Differentially expressed genes of *Scenedesmus* sp. BHU1 under salinity stress (0.4 M NaCl).

| **Transcript_id** | **Metabolic pathway** | **Protein** | **Expression changes** |
| --- | --- | --- | --- |
| TRINITY_DN155_c0_g1 | Photosynthesis | Photosystem I P700 chlorophyll a apoprotein A2 (psaB) | Up |
| TRINITY_DN205_c0_g1 |  | Photosystem I P700 chlorophyll a apoprotein A1 (psaA) | Up |
| TRINITY_DN363_c0_g1 |  | Photosystem I iron-sulfur center | Up |
| TRINITY_DN10281_c0_g1 |  | UDP-sulfoquinovose synthase (chloroplastic) | Down |
| TRINITY_DN1031_c1_g2 |  | Chlorophyll a-b binding protein 4, chloroplastic (LHCA4) | Down |
| TRINITY_DN35221_c2_g1 |  | Chlorophyll a-b binding protein of LHCII type I, chloroplastic | Down |
| TRINITY_DN1038_c0_g1 |  | Ribulose bisphosphate carboxylase small subunit (chloroplastic 2) | Down |
| TRINITY_DN12112_c0_g3 |  | ATP synthase subunit alpha (atpA) | Down |
| TRINITY_DN12112_c0_g2 |  | ATP synthase subunit delta (atpH) | Down |
| TRINITY_DN12112_c0_g1 |  | ATP synthase subunit alpha (atpG) | Down |
| TRINITY_DN12754_c0_g1 |  | Chlorophyll a-b binding protein 7, chloroplastic (LHCQ) | Down |
| TRINITY_DN132_c0_g1 |  | Chlorophyll a-b binding protein 6, chloroplastic (LHCA1) | Down |
| TRINITY_DN132_c0_g5 |  | Chlorophyll a-b binding protein type 2 member 1A, chloroplastic | Down |
| TRINITY_DN13965_c0_g4 |  | Chlorophyll a-b binding protein 1D (Lhcb2-1) | Down |
| TRINITY_DN20973_c0_g1 |  | Chlorophyll a-b binding protein CP29 (LHCB4) | Down |
| TRINITY_DN2194_c0_g1 |  | Photosystem I chlorophyll a/b-binding protein 5, chloroplastic (LHCA) | Down |
| TRINITY_DN21978_c0_g3 |  | Chlorophyll a-b binding protein of LHCII type I, chloroplastic (LHCB5) | Down |
| TRINITY_DN21978_c0_g2 |  | Chlorophyll a-b binding protein CP26, chloroplastic (LHCB5) | Down |
| TRINITY_DN28429_c0_g1 |  | Chlorophyll a-b binding protein P4, chloroplastic (LHCA5) | Down |
| TRINITY_DN286282_c0_g1 |  | Photosystem I chlorophyll a/b-binding protein 3-1, chloroplastic (LHCA3) | Down |
| TRINITY_DN28824_c1_g1 |  | Chlorophyll a-b binding protein 8, chloroplastic (LHCA3) | Down |
| TRINITY_DN36987_c2_g1 |  | Chlorophyll a-b binding protein 1B-21, chloroplastic (LHCA5) | Down |
| TRINITY_DN43560_c0_g1 |  | Chlorophyll a-b binding protein 3, chloroplastic (LHCA3) | Down |
| TRINITY_DN7_c0_g2 |  | Photosystem I chlorophyll a/b-binding protein 5, chloroplastic (LHCA5) | Up |
| TRINITY_DN23016_c0_g1 |  | Oxygen-evolving enhancer protein 1, chloroplastic (psbO) | Down |
| TRINITY_DN248437_c0_g1 |  | PsbP domain-containing protein 7, chloroplastic (psbP5) | Down |
| TRINITY_DN1392_c1_g1 |  | PsbP-like protein 1, chloroplastic (psbP3) | Down |
| TRINITY_DN65_c0_g1 |  | Oxygen-evolving enhancer protein 3, chloroplastic (psbQ) | Down |
| TRINITY_DN12990_c0_g2 |  | Photosystem I reaction center subunit N, chloroplastic (PsaN) | Up |
| TRINITY_DN119_c3_g3 |  | Photosystem II protein PSBS1 (PSBS) | Down |
| TRINITY_DN178_c0_g2 |  | Ferredoxin, chloroplastic (petF3) | Down |
| TRINITY_DN120330_c1_g1 |  | Photosystem II protein D1 (psbA) | Down |
| TRINITY_DN121725_c0_g1 |  | Photosystem II D2 protein (psbD) | Down |
| TRINITY_DN1540_c0_g1 |  | Photosystem II repair protein PSB27-H1, chloroplastic | Down |
| TRINITY_DN16863_c0_g2 |  | Photosystem II reaction center proteins PsbY, chloroplastic (psbY) | Down |
| TRINITY_DN17181_c0_g1 |  | Photosystem II reaction center Psb28 protein | Down |
| TRINITY_DN19139_c0_g1 | Photosynthetic carbon fixation | Phosphoribulokinase, chloroplastic (PRK) | Down |
| TRINITY_DN12198_c1_g2 |  | Transketolase (TKTA) | Down |
| TRINITY_DN305194_c0_g1 |  | Fructose-1,6-bisphosphate aldolase/phosphatase (fbp) | Down |
| TRINITY_DN34186_c0_g1 |  | Fructose-1,6-bisphosphatase class 1 (fbp) | Up |
| TRINITY_DN11916_c0_g2 |  | Glyceraldehyde-3-phosphate dehydrogenase (gapA) | Down |
| TRINITY_DN72_c0_g3 |  | Sedoheptulose-1,7-bisphosphatase, chloroplastic (SBPase) | Down |
| TRINITY_DN878_c0_g1 |  | Ribulose bisphosphate carboxylase large chain (rbcL) | Down |
| TRINITY_DN136953_c0_g1 | Porphyrin and chlorophyll metabolism | Oxygen-dependent coproporphyrinogen-III oxidase (CPOX) | Down |
| TRINITY_DN12686_c0_g1 |  | Porphobilinogen deaminase, chloroplastic (HEMC) | Down |
| TRINITY_DN10133_c0_g1 |  | Chlorophyll synthase, chloroplastic (chlG) | Down |
| TRINITY_DN144107_c0_g1 |  | Chlorophyllide a oxygenase, chloroplastic (CAO) | Down |
| TRINITY_DN16140_c2_g1 |  | Protochlorophyllide reductase, chloroplastic (PORA) | Down |
| TRINITY_DN14191_c0_g1 | Glycolysis | Hexokinase-1 (HK) | Up |
| TRINITY_DN33334_c0_g2 A |  | ATP-dependent 6-phosphofructokinase (PFK) | Up |
| TRINITY_DN141600_c0_g1 |  | Fructose-bisphosphate aldolase (ALDO) | Down |
| TRINITY_DN11916_c0_g1 |  | Phosphoglycerate kinase (PGK) | Down |
| TRINITY_DN147785_c0_g1 |  | Enolase (eno) | Up |
| TRINITY_DN125045_c0_g1 |  | Pyruvate dehydrogenase E1 component (aceE) | Up |
| TRINITY_DN125461_c0_g1 |  | Pyruvate dehydrogenase E1 component subunit alpha (pdhA) | Up |
| TRINITY_DN1257_c0_g1 |  | Pyruvate dehydrogenase E1 component subunit beta (pdhB) | Down |
| TRINITY_DN182244_c0_g1 |  | Pyruvate kinase (PK) | Up |
| TRINITY_DN209450_c0_g1 | Tricarboxylic acid cycle | Citrate synthase 1 (citA) | Up |
| TRINITY_DN104933_c0_g1 |  | Isocitrate dehydrogenase kinase/phosphatase (aceK) | Up |
| TRINITY_DN1555_c0_g1 |  | Malate dehydrogenase (mdh) | Up |
| TRINITY_DN115201_c0_g1 |  | 2-oxoglutarate dehydrogenase E1 component (sucA) | Up |
| TRINITY_DN124403_c0_g1 |  | Fumarate hydratase class II (fumC) | Up |
| TRINITY_DN180211_c0_g1 |  | Fumarate hydratase class II (merA) | Up |
| TRINITY_DN238383_c0_g1 |  | Fumarate hydratase class II 2 (fumC) | Up |
| TRINITY_DN238700_c0_g1 |  | Fumarate hydratase class II 2 (yigZ) | Up |
| TRINITY_DN1481_c0_g1 | Starch metabolism | Alpha-amylase type B isozyme (AMY1) | Up |
| TRINITY_DN213032_c0_g1 |  | Starch synthase 3, chloroplastic/amyloplastic | Up |
| TRINITY_DN248043_c0_g1 |  | Glycogen synthase (glgA) | Up |
| TRINITY_DN121408_c0_g1 |  | Trehalose/maltose transport system permease protein MalF | Up |
| TRINITY_DN161169_c0_g1 |  | Beta-amylase | Up |
| TRINITY_DN143286_c0_g1 |  | Glucose-1-phosphate adenylyltransferase (glgC) | Up |
| TRINITY_DN30851_c0_g1 | Sucrose metabolism | Sucrose synthase 2 (SUS4) | Up |
| TRINITY_DN100788_c0_g1 |  | UDP-glucose 6-dehydrogenase (ugd) | Up |
| TRINITY_DN103144_c0_g1 | Lipid accumulation | Acetoacetyl-CoA synthetase (acsA) | Up |
| TRINITY_DN2356_c0_g2 |  | Pyruvate dehydrogenase E1 component (pdhR) | Up |
| TRINITY_DN143485_c0_g1 |  | Malonyl CoA-acyl carrier protein transacylase (fabD) | Down |
| TRINITY_DN129139_c0_g1 |  | Biotin carboxyl carrier protein of acetyl-CoA carboxylase (accB) | Up |
| TRINITY_DN172364_c0_g1 |  | Beta-ketoacyl-[acyl-carrier-protein] synthase III (fabH) | Up |
| TRINITY_DN120442_c0_g1 |  | 3-oxoacyl-[acyl-carrier-protein] synthase 2 (fabF) | Up |
| TRINITY_DN109304_c0_g1 |  | 3-oxoacyl-[acyl-carrier-protein] synthase 1 (fabB) | Up |
| TRINITY_DN1131_c2_g1 |  | 3-oxoacyl-[acyl-carrier-protein] reductase FabG (fabG) | Up |
| TRINITY_DN118740_c0_g1 |  | 3-hydroxyacyl-[acyl-carrier-protein] dehydratase (babZ) | Up |
| TRINITY_DN20838_c0_g1 |  | Long chain acyl-CoA synthetase 4 (ACSL) | Up |
| TRINITY_DN59307_c1_g2 |  | Very-long-chain (3R)-3-hydroxyacyl-CoA dehydratase 2 | Up |
| TRINITY_DN19955_c0_g2 |  | 3-ketoacyl-CoA synthase 16 | Up |
| TRINITY_DN245323_c0_g1 |  | 3-ketoacyl-CoA synthase 7 | Up |
| TRINITY_DN10372_c2_g1 |  | stearoyl-CoA desaturase (Delta-9 desaturase) (DesC) | Up |
| TRINITY_DN221768_c0_g1 | Cellulose Biosynthesis | Cellulose synthase catalytic subunit [UDP-forming] (bcsA) | Up |
| TRINITY_DN178333_c0_g1 |  | Cellulose synthase operon protein C (bcsC) | Up |

**Supplementary Figures and their captions**

**
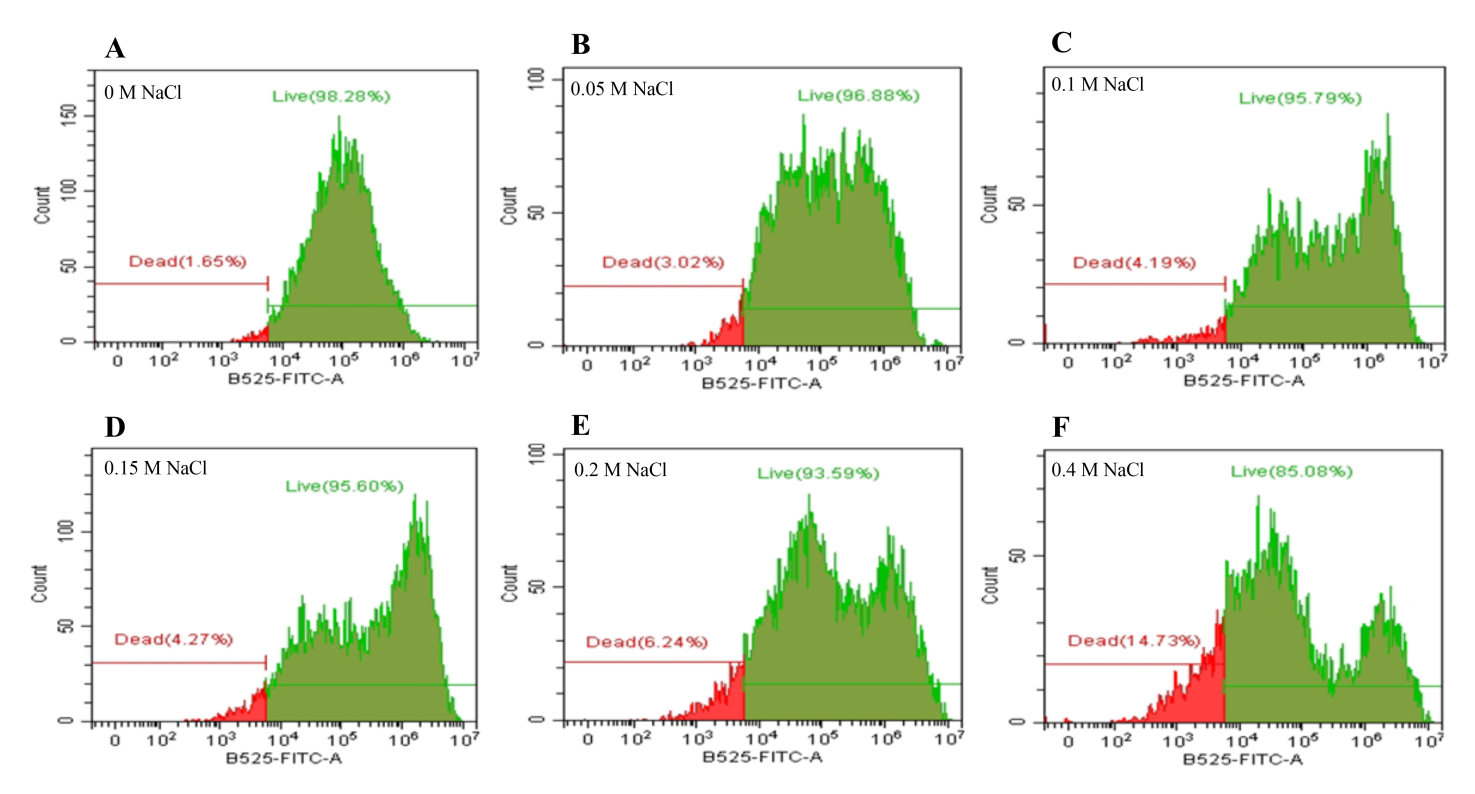
**

**Supplementary Figure S1.** Flow cytometry (FCM) plots of *Scenedesmus* sp. BHU1 cells were used to assay viability under different concentrations of NaCl.


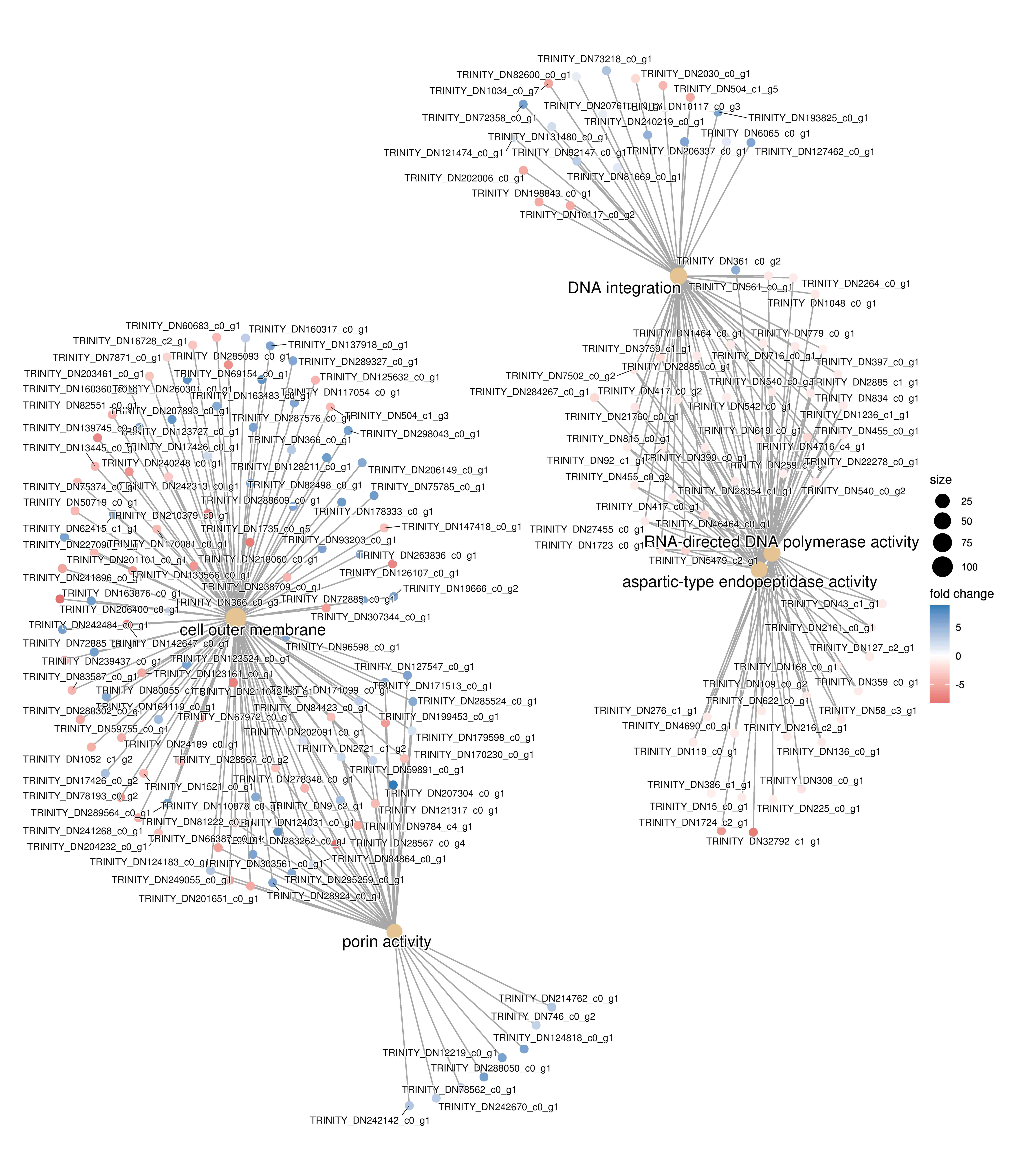


**Supplementary Figure S2.** Network plot displaying the gene ontology for differentially expressed genes in *Scenedesmus* sp. BHU1 (control vs. stressed; go ora).


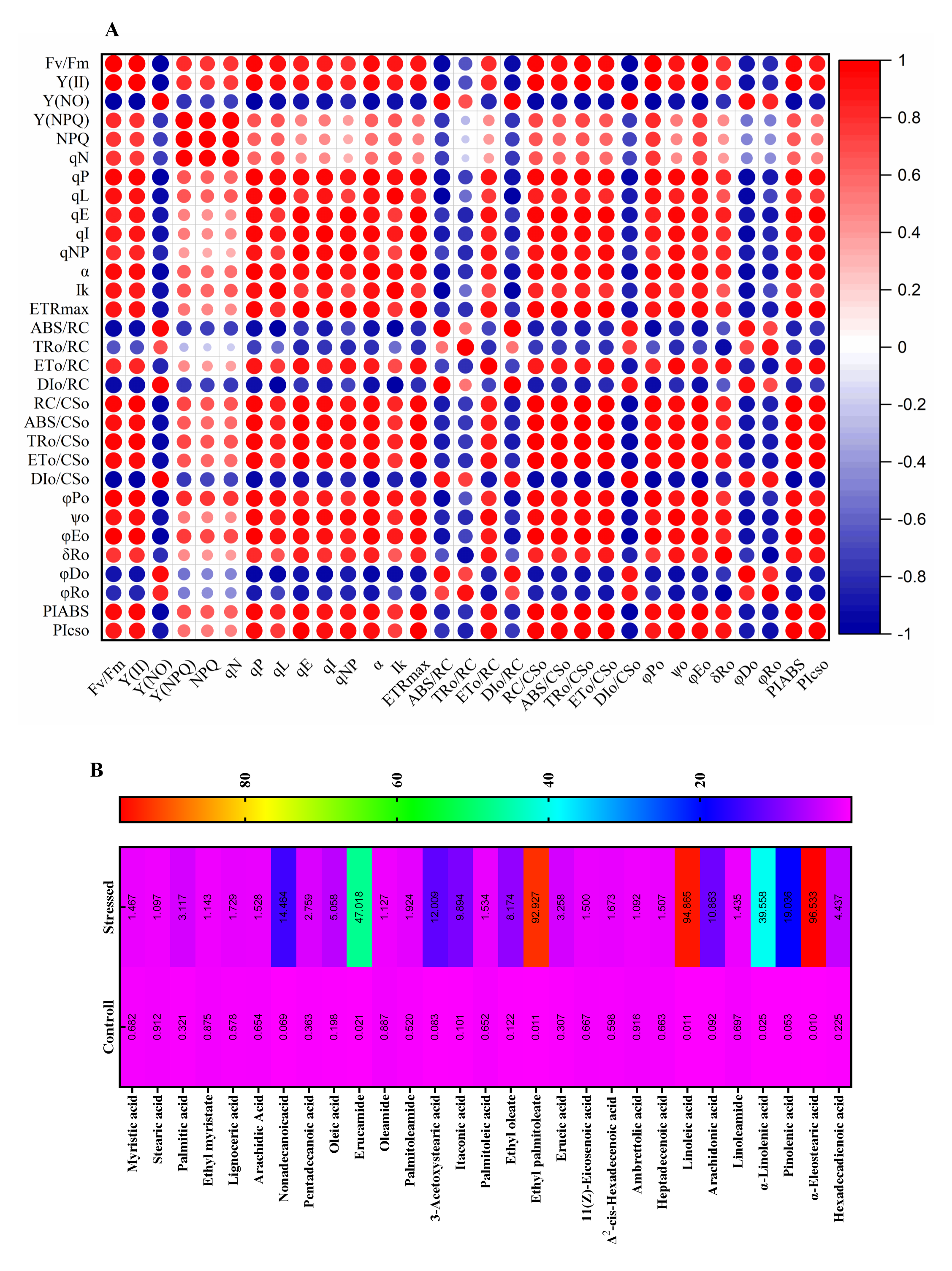


**Supplementary Figure S3.** Panel **(A)** represents: Pearson Correlation plot between the different photosynthetic parameters (slow kinetics, rapid light curve, and fast kinetics) in *Scenedesmus* sp. BHU1 cells subjected to control and stressed conditions. Red color circle showed perfect positive correlation and blue circle showed perfect negative correlation with a gradient of color showing in between correlation values. On the other hand panel **(B)** represents: heat map showed lipidomics in the form of fatty acid profiling of control and stressed *Scenedesmus* sp. BHU1 grown cells.
